# Impact of m^6^A modification and transcript quantity on mRNA composition in plant stress granules under hypoxia

**DOI:** 10.1093/jxb/eraf046

**Published:** 2025-02-05

**Authors:** Dawid Jakub Kubiak, Michał Wojciech Szcześniak, Karolina Ostrowska, Dawid Bielewicz, Susheel Sagar Bhat, Katarzyna Niedojadło, Zofia Szweykowska-Kulińska, Artur Jarmołowski, Rupert George Fray, Janusz Niedojadło

**Affiliations:** Department of Cellular and Molecular Biology, Nicolaus Copernicus University, Lwowska 1, 87-100, Torun, Poland; Institute of Human Biology and Evolution, Faculty of Biology, Adam Mickiewicz University, Poznan 61-614, Poland; Department of Cellular and Molecular Biology, Nicolaus Copernicus University, Lwowska 1, 87-100, Torun, Poland; Centre for Advanced Technologies, Adam Mickiewicz University, Poznan 61-614, Poland; Department of Gene Expression, Institute of Molecular Biology and Biotechnology, Faculty of Biology, Adam Mickiewicz University, Poznan 61-614, Poland; Department of Gene Expression, Institute of Molecular Biology and Biotechnology, Faculty of Biology, Adam Mickiewicz University, Poznan 61-614, Poland; Department of Cellular and Molecular Biology, Nicolaus Copernicus University, Lwowska 1, 87-100, Torun, Poland; Department of Gene Expression, Institute of Molecular Biology and Biotechnology, Faculty of Biology, Adam Mickiewicz University, Poznan 61-614, Poland; Department of Gene Expression, Institute of Molecular Biology and Biotechnology, Faculty of Biology, Adam Mickiewicz University, Poznan 61-614, Poland; School of Biosciences, The University of Nottingham, Sutton Bonington Campus, Leicestershire LE12 5RD, UK; Department of Cellular and Molecular Biology, Nicolaus Copernicus University, Lwowska 1, 87-100, Torun, Poland; Michigan State University, USA

**Keywords:** *Arabidopsis thaliana*, hypoxia, *Lupinus*, m^6^A modification, stress granules, transcriptome

## Abstract

Stress granules (SGs) are cytoplasmic structures that appear in response to unfavorable environmental conditions. The mechanisms governing the accumulation of transcripts in SGs are only partially understood, and despite the recognized role of N^6^-methyladenosine (m^6^A) in plant transcriptome regulation, its impact on SG composition and assembly remains unknown. In *Lupinus angustifolius*, SGs display a distinctive bi-zonal structure comprising of a ring and a central area with differences in their ultrastructure and composition. Following a transcriptome analysis, specific mRNAs were chosen to investigate their localization within SGs and to assess m^6^A levels. Transcripts of hypoxia-responsive genes (*ADH1* and *HUP7*) showed significantly lower levels of m^6^A compared to housekeeping genes, but only *ADH1* was absent in SGs. *HUP7* mRNA, characterized by a low quantity of m^6^A, was present both in the SGs and the cytoplasm, probably due to an extremely high expression level. The m^6^A modification was observed only during the assembly of SGs. In mutants of Arabidopsis with reduced levels of m^6^A, ECT2 (a reader of m^6^A) was not observed in SGs, and poly(A) RNA levels and the number of SGs were reduced. Our findings thus demonstrate a limited impact of m^6^A modification on SG assembly; however, the interplay between m^6^A modification and the overall transcript quantity in the cytoplasm appears to play a regulatory role in mRNA partitioning and assembly of SGs.

## Teaser

m^6^ A modification and transcript abundance in cells regulate the number of stress granules and the quantity of poly(A) they contain in plants under hypoxia, and control selective mRNA partitioning into the granules.

## Introduction

The response of a cell to adverse environmental conditions requires reprogramming of almost every aspect of the RNA lifecycle. It has been shown that the spatial regulation of this process during stress is important (de Nadal *et al.*, 2011). The structural manifestation of this phenomenon is the storage of RNA in stress granules (SGs) in the cytoplasm. The mRNAs accumulated in SGs are separated from the translation complexes, and this mechanism enables the reduction of protein synthesis and regulates the selectivity of the translation process. The RNA accumulated within the SGs structures can be utilized following the end of stress (Kearly *et al.*, 2022).

SGs are dynamic membrane-less cytoplasmic structures that form in plant and animal cells exposed to abiotic and biotic stress (Maruri-López *et al.*, 2021). It is commonly believed that stress-induced inhibition of translation serves as a key trigger for the formation of SGs (Sorenson and Bailey-Serres 2014; Hofmann *et al.*, 2021). Previous studies in mammals and yeast suggest that a multi-step mechanism, conserved among eukaryotes and driven by liquid–liquid phase separation (LLPS), is responsible for the formation and dynamics of SGs. Although information on SG formation in plants is limited, several studies have provided evidence supporting the same mechanism as proposed for animal cells (Gutierrez-Beltran *et al.*, 2021; Allen and Strader 2022).

The first step in the formation of SGs is nucleation, during which a high concentration of messenger ribonucleoproteins (mRNPs) and a combination of protein–protein, RNA–RNA, and protein–RNA interactions induces LLPS of mRNP complexes, leading to the assembly of the SG cores (Niewidok *et al.*, 2018). During core growth, the secondary structure of RNA is of particular importance and can act as a binding factor to liquid condensates; however, the transcriptome of SGs and the role of RNA structure in their formation remain poorly understood in plants. The microscale SGs then combine to form a multicore structure immersed in a single shell (Wheeler *et al.*, 2016); again, however, the distinction between cores and shells in plants is much less obvious compared to animals. The final step in SG formation is shell condensation (Markmiller *et al.*, 2018).

It has been shown that SGs might be a storage site for translation-ready mRNA not associated with ribosomes under stress conditions. Surviving abiotic stress is related to limiting energy-intensive processes in the cell (Das *et al.*, 2022), and therefore reducing the intensity of translation becomes necessary under abiotic stress conditions. Nonetheless, the cellular response to stressful conditions requires the presence of specific proteins, but the exact mechanisms governing selective translation remain unclear (Merchante *et al.*, 2017). The separation of mRNA molecules from the translational apparatus and their sequestration in SGs might serve as a strategy to enable the selective translation of specific proteins during stress conditions (Hofmann *et al.*, 2021); however, the involvement of SGs in the regulation of the translation process has not yet been clearly determined. The formation of SGs appears to be a consequence of translation inhibition rather than its cause. In cells depleted in GTPase-activating protein-binding protein (G3BP)1/2, which do not form SGs, it has been shown that translation is still inhibited during stress (Kedersha *et al.*, 2016; Mateju *et al.*, 2020).

 In addition, studies have indicated the absence of the 60S ribosomal subunit in SGs. Localization studies of the 60S ribosomal proteins L37 and L5 have shown that they are excluded from SGs (Kimball *et al.*, 2003). Nevertheless, Mateju *et al.* (2020) have shown in animal cells that SG-associated translation is not rare, and translating mRNAs can be observed transitioning between the cytosol and the SGs without changing their translational status. Further research is needed in animal cells, and similar investigations should be undertaken in plants.

A new layer of regulation of gene expression has become apparent in recent years, highlighting the involvement of nucleotide modification in mRNA, a phenomenon termed epitranscriptomic regulation. The most common modification in mRNA is N^6^-methyladenosine (m^6^A) (Anderson *et al.*, 2018); however, its role in regulating transcript localization is not fully understood. Anders *et al.* (2018) showed that in animal cells under conditions of oxidative stress, the level of m^6^A increased in individual transcripts, and the total amount of methylated transcripts also increased. Interestingly, 96% of the transcripts in which m^6^A was up-regulated in response to stress were located in the SGs. The removal of a methylation site in a single transcript resulted in it not being targeted to the SGs. Ries *et al.* (2023) obtained similar results in a knockout cell line of the *METTL3* gene (responsible for methylation), indicating that long mRNAs accumulated in the SGs due to m^6^A methylation. An increasing body of evidence links m^6^A modifications and YTH-domain-containing proteins to SG formation (Sun *et al.*, 2023). The role of m^6^A in plants remains less well studied, with fewer publications addressing this topic (Scutenaire *et al.*, 2018; Prall *et al.*, 2023). It has been shown that methylation of the heat-activated retrotransposon Onsen in Arabidopsis leads to its localization in SGs, thus suppressing its mobility (Fan *et al.*, 2023). The potential role of m^6^A modifications in mRNA localization in SGs might be evidenced by the fact that YTH proteins, responsible for m^6^A recognition, have been found in Arabidopsis SGs (Scutenaire *et al.*, 2018; Kosmacz *et al.*, 2019). In animals, knockout of *YTHDF3* resulted in the loss of the m^6^A signal being co-localized with SGs (Anders *et al.*, 2018). Whether these proteins and m^6^A levels affect transcript sequestration into SGs in plants remains to be determined.

In this study, we identified SGs with a bi-zonal morphology that formed under hypoxia stress, and we investigated the roles of epitranscriptomic mechanisms in the functioning of SGs.

## Materials and methods

### Plant growth and treatments

Seeds of *Lupinus angustifolius* (lupin) cv. Sonet were surface-sterilized by soaking them in 70% (v/v) ethanol for 5 min, followed by rinsing five times with sterile, deionized water. The seeds were then soaked in water for 30 min and germinated for 3 d at 21 °C on water-soaked tissue paper in the dark in a growth chamber. Seedlings with roots of 3–4 cm were then selected and different periods of hypoxia were induced by submerging the plants in containers filled with tap water (oxygen concentration 8.7 mg l^–1^) at 21 °C in the dark. The water level was maintained ~4 cm above the plants. For reoxygenation after hypoxia, the plants were returned to water-soaked tissue paper at 21 °C in the dark.

Translation in lupin roots was inhibited using 4 mg ml^–1^ cycloheximide (Merck). After 14 h of hypoxia, the seedlings were immersed in water containing the inhibitor and transferred to a vacuum chamber for 1 h. Following this, some of the roots were sampled for fixation (described below), while the remaining seedlings were transferred to tissue paper soaked in the inhibitor in a growth chamber at 21 °C in the dark for either 6, 10, or 20 h before the roots were sampled.

The *Arabidopsis thaliana* ecotype Col-0 the *ECT2::GFP* and *RBP47::GFP* lines were utilized in this study. In addition, the *pABI3::MTA-mta* line (the *mta* mutant transformed with *MTA* under the control of the *ABI3* promoter) was used (Bodi *et al.*, 2012). Furthermore we performed crosses to obtain the lines *ABI3::MTA* × *RBP47b::GFP* and *ABI3::MTA* × *ECT2::GFP*. Arabidopsis seeds were surface-sterilized first in 75% ethanol for 2 min and then in 5% sodium hypochlorite for 8 min, followed by seven rinses with sterile distilled water. After surface-sterilization, the seeds were sown on plates containing 2.15 g l^–1^ Murashige and Skoog (MS) medium (Duchefa Biochemie), supplemented with 1 ml of MS ×1000 concentrated vitamin mixture (103.1 mg l^–1^; Duchefa Biochemie), 10 g l^–1^ sucrose, and 7.5 g l^–1^ agar, pH 5.8, and were incubated at 4 °C for 2 d in the dark. The seedlings were then cultivated in a growth chamber under control conditions of 21 °C under long days (16/8 h light/dark) at 150 µmol m^–2^ s^–1^). For the hypoxia treatment, the MS plates containing 2-week-old seedlings were submerged for 3 d in a container filled with sterile water (oxygen concentration 8.7 mg l^–1^, ) with the water level ~5 cm above the seedlings, and cultivated in a growth chamber at 21 °C under low light intensity (50 µmol m^–2^ s^–1^).The seedlings were then removed from the water and grown under the control conditions.

### Enrichment of cytoplasmic stress granules

Enrichment of the cytoplasmic SGs was performed using the method described previously by Lei *et al.* (2021). Briefly, 2 g of frozen lupin roots was ground in liquid nitrogen using a chilled mortar and pestle, and the material was resuspended in 5 ml of lysis buffer [50 mM Tris-HCl, pH 7.4, 100 mM KOAc, 2 mM MgOAc, 0.5% NP-40, 0.5 mM DTT, Protease Inhibitor Cocktail (Sigma), 1 U μl^–1^ RNasin Plus RNase Inhibitor (Promega)]. The resulting slurry was filtered through two layers of Miracloth and centrifuged at 850 *g* for 5 min at 4 °C. The supernatant was transferred to a new tube and 5 ml of lysis buffer was added. The samples were centrifuged at 4000 *g* for 10 min at 4 °C. The supernatant was removed, and the pellet was resuspended in 2 ml of lysis buffer. The samples were centrifuged at 18 000 *g* for 10 min at 4 °C, after which the supernatant was removed and the pellet was resuspended in 1 ml of lysis buffer. Finally, following centrifugation at 850 *g* for 10 min at 4 °C, the supernatant with enriched SG fraction was transferred to a new 1.5 ml microcentrifuge tube.

### *In situ* hybridization and double-labeling of m^6^A and Poly(A) Binding Protein 2

To prepare lupin protoplasts, root meristems were excised and fixed in 4% formaldehyde (Polysciences) in PBS (pH 7.2) for 24 h at 4 °C. After washing, the fixed root tips were placed in citric acid-buffered digestion solution (pH 4.8) containing 5% cellulase (Onozuka R-10) and 35 U ml^–1^ pectinase (Sigma) for 2 h at 35 °C. Following rinsing with PBS, the root tips were squeezed onto slides.

To prepare semi-thin sections for both lupin and Arabidopsis, the fixed roots were dehydrated in increasing concentrations of ethanol containing 10 mM dithiothreitol (DTT) (ThermoFisher Scientific). They were then embedded in BMM resin (butyl methacrylate, methyl methacrylate, 0.5% benzoyl ethyl ether with 10 mM DTT; Merck) at –20 °C under UV light for polymerization. The sections were cut on a Leica UCT ultramicrotome into 1.5 µm sections.

After a 1 h pre-hybridization step, hybridization was performed for at least 12 h at 26 °C in a hybridization buffer [50% (v/v); Merck] containing 30% (v/v) formamide (Merck) and 1:250 dilution of probes. For fluorescence *in situ* hybridization (FISH), probes targeting the central mRNA sequence of *ADH1*, *HUP7*, *PCO1*, *L37*, *L44*, *RPB1*, as well as the *18S* and *26S* rRNAs (representing the small and large ribosomal subunits) and the 5'-UTR sequence of *RPB1* were used (sequences of probes are given in Supplementary Table S1). For double-labeling of poly(A) RNA and selected mRNAs, two probes were applied simultaneously. The sections were washed in 2× SSC, and nuclei were stained with Hoechst 33342 (ThermoFisher Scientific).

For simultaneous localization of RNA and proteins, incubations were performed with primary rabbit antibodies against N^6^-methyladenosine (NEB) or Poly(A) Binding Protein 2 (PAB2; courtesy of Cecile Bousquet-Antonelli and Rémy Merret, CNRS, France), diluted to 1:200 and 1:100, respectively, in PBS (pH 7.2) with 1% BSA overnight at 4 °C. The slides were then washed in PBS and incubated for 1 h at 35 °C with secondary antibodies diluted to 1:250 in PBS buffer with 1% BSA (Alexa Fluor 488-labeled mouse anti-rabbit IgG; Molecular Probes). After washing twice in 2× SSC, the *in situ* hybridization was performed as described above. For control reactions, probes and primary antibodies were omitted. An additional immunocytochemical control reaction for m^6^A was performed, where the sections were treated with RNase A for 30 min prior to the reaction.

### Microscopic analysis and quantitative measurement of fluorescence signals

To determine the fluorescence signal obtained from the poly(A) RNA *in situ* hybridization and the diameter of the SGs, at least 60 cells from three different experiments were analysed. Between 30–60 cells were analysed for each transcript. For quantitative measurements, each experiment was performed using constant temperatures, incubation times, and concentrations of probes and antibodies. In addition, all images were acquired under constant acquisition conditions (laser power, emission band, gain, and resolution). The results were recorded using an Olympus FV3000 microscope and FV3000 software, equipped with a DIC H immersion objective with 63× magnification (numerical aperture 1.4) and exposure of 4 Hs. Images were acquired sequentially in red (Cy3), green (Alexa Fluor 488), and blue (Hoechst 33342) channels. Optical sections were collected at 0.5 um intervals. Analysis and processing of the obtained images was carried out using the ImageJ software. The fluorescence threshold in the sample was determined based on the autofluorescence of the control reaction that was collected under the same conditions.

The signal intensity per cytoplasm/SG image was measured and expressed in relative fluorescence units or fluorescence signal per /um^2^ of the analysed structure. The measurement of fluorescence intensity for the studied transcripts was performed in SGs and in the cytoplasm in three arbitrarily chosen locations each with a size corresponding to the dimensions of the SGs for each cell.

### Immunogold labeling of PAB2

For immunogold labeling, roots were fixed in a solution containing 4% formaldehyde (v/v) and 0.25 % (v/v) glutaraldehyde (both Polysciences) in PBS (pH 7.2) for 24 h at 4 °C and then dehydrated through a graded series of ethanol. The material was embedded in LR Gold resin (Sigma) according to the standard protocol. The ultrathin sections were incubated first with anti-PAB2 rabbit primary antibody diluted 1:100 in 1% BSA in PBS overnight at 4 °C and then with goat anti-rabbit IgG 20 nm gold-conjugated secondary antibody (BBInternational) diluted 1:50 in 1% BSA in PBS for 1 h at 37 °C. Finally, the material was stained with 2.5 % uranyl acetate and 0.4% lead citrate solutions, and examined using a JEOL 1010 TEM at 80 kV. For a negative control, the primary antibody was omitted.

### Electron microscopy

For standard TEM, roots at 4–5 mm long were fixed in 3% glutaraldehyde (Polysciences) in PBS (pH 7.2) overnight at 4 °C. After washing in PBS, the roots were post-fixed in 1% OsO4 in PBS buffer for 1 h at 4 °C. The material was then dehydrated in increasing concentrations of ethanol and embedded in Spurr resin (Sigma). Ultrathin sections were placed on copper grids, contrasted in 2.5% lead citrate and 2.5 % uranyl acetate (20 min each), and examined using the JOEL 1010 TEM.

### Single-molecule localization microscopy

A Bruker Vutara VXL microscope with widefield illumination and bi-plane detection for 3D acquisitions was used to analyse the distribution of individual molecules of poly(A) RNA on semithin sections of lupin roots using stochastic optical reconstruction microscopy (STORM). The following parameters were used: 20 ms exposure time; 90% 638 nm laser power; 692/85 nm emission filter for Cy5 channel; and 50 background threshold applied in the localization algorithm. Cluster analysis was performed using the DBSCAN cluster algorithm with the following parameters: 0.3 µm maximum particle distance; 50 minimum particle count to form a cluster; and a 50 nm isosurface particle size.

### RNA isolation and m^6^A-RNA immunoprecipitation (MeRIP)

Total RNA was isolated from 100 mg of roots using TRIzol reagent (ThermoFisher Scientific) and a Direct-zol RNA MiniPrep Kit (Zymo Research).

For immunoprecipitation of m^6^A-RNA, total RNA was extracted from the meristematic parts of the lupin roots as described above, followed by poly(A) RNA enrichment using PolyATtract^®^ mRNA Isolation Systems (Promega). A 9 μg sample of polyA RNA (1 μg μl^–1^) was used for immunoprecipitation, which was performed with an EpiMark N^6^-Methyladenosine Enrichment Kit (NEB) according to the manufacturer’s instructions. Briefly, 25 μl of magnetic Dynabeads Protein G for Immunoprecipitation (ThermoFisher Scientific) were washed with 250 μl of reaction buffer. Next, 2 μl of anti-m^6^A antibody per sample was incubated with the beads for 1 h at 4 °C with shaking at 4 rpm. The beads were then washed twice with reaction buffer and resuspended in 250 ul of reaction buffer. A 9 µg sample of poly(A) RNA was spiked in with 1 μl of positive and negative control from the kit (1:1000 dilution) and made up to 13 μl final volume with nuclease-free water. As input, 10% of the sample volume (1.3 µl) was taken and the residue was added to the beads. Then, 3 µl of RNasin Ribonuclease Inhibitor (Promega) was added to the beads, followed by incubation for 2 h at 4 °C with shaking at 3 rpm. After incubation, the beads were washed twice with reaction buffer, LSB buffer (0.02 M Tris HCl, pH 8, 0.002 M EDTA, 1% Triton X-100, 0.15 M NaCl, 0.1% SDS), and HSB buffer (0.02 M Tris HCl, pH 8, 0.002 M EDTA, 1% Triton X-100, 0.5 M NaCl, 0.1% SDS). RNA was then extracted using acid phenol:chloroform (5:1, pH 4.5),

### Quantitative real-time PCR

Reverse transcription was performed using a Transcriptor First Strand cDNA Synthesis Kit (Roche) with oligo d(T) primers, according to the manufacturer’s protocol. The samples were incubated in a thermocycler at 25 °C for 10 min, then 55 °C for 30 min, and finally 5 min at 85 °C (C1000 Touch Thermal Cycler; Bio-Rad).

Real-time PCR was performed using a LightCycler 480 SYBR Green Master Kit (Roche). The sequences of the primers used are shown in Supplementary Table S1. The thermal profile was carried out according to default parameters of a pre-incubation step at 95 °C for 10 min (1 cycle) and an amplification step at 95 °C for 10 s, 52 °C for 10 s, and 72 °C for 20 s (45 cycles). The PCR was performed on three independent biological samples with three technical replicates. *Ubiquitin C* (*UBC*) and *Helicase* (*HEL*) have both been shown to be stable reference genes in *Lupinus angustifolius* (Taylor *et al.*, 2016), and we selected *UBC* as the reference in this study. The amount of transcripts was calculated using the 2^−ΔΔ*C*T^ method (Livak and Schmittgen, 2001). To quantify the changes in m^6^A modification of the genes of interest, MeRIP enrichment followed by quantitative real-time PCR was performed. Briefly, after MeRIP, all MeRIP RNA and input RNA samples were subjected to qRT–PCR. The amount of methylated transcripts level was determined by qRT-PCR using the 2^–Δ*C*T^ methodology, and the Δ*C*_T_ value (cycle threshold) was normalized by Δ*C*_T,IP_, Δ*C*_T,input_, and the input dilution factor. To determine the PCR efficiencies, standard curves for target and control genes were obtained using a series of cDNA dilutions as a template.

### Library preparation and sequence analysis

Poly(A) RNA of the highest quality from three biological replicates of roots from each treatment (normoxia, hypoxia, and hypoxia followed by reoxygenation) was used for the preparation of cDNA libraries for next generation sequencing (NGS) using a NEBNext Ultra II RNA Library Prep Kit for Illumina according to the manufacturer’s protocol. Briefly, ~5 ng of poly(A) RNA was fragmented for 7 min, followed by cDNA synthesis, end repair, and adaptor ligation. The yield of amplified libraries was measured on a Qubit 4 fluorometer using a Qubit dsDNA Quantification Assay Kit (High Sensivity) (ThermoFisher Scientific). The libraries were sequenced by Genomed (Warsaw, Poland) using an Illumina NovaSeq6000 in PE150 mode, with 20 million paired reads per sample.

### Computational analysis of RNA-sequencing data

The quality of raw sequencing reads was assessed with FastQC v0.11.5. Quality trimming, filtering, and adapter clipping were done using BBDUK2 v37.02 from the BBMAP package. rRNA-mapping reads were discarded using Bowtie 2 v2.3.5.1. The *L. angustifolius* ribosomal RNAs came from Ensembl Plants 56. The clean reads were then used for an *ab initio* transcriptome assembly with StringTie v1.3.3b and the lupin genome LupAngTanjil_v1.0. The assembled transcriptome was compared to a reference transcriptome, LupAngTanjil_v1.0.55, obtained from ENSEMBL Plants 55 (Yates *et al.*, 2022), using Cuffcompare v2.2.1 (Trapnell *et al.*, 2012) to identify previously annotated transcripts. To further characterize the transcriptome, known orthologs in Arabidopsis were retrieved from ENSEMBL Plants. ORFs and encoded proteins were predicted using TransDecoder v5.0.2 (Haas *et al.*, 2013). The predicted protein sequences were then subjected to a BLASTP v2.9.0+ search (Camacho *et al.*, 2009) against the SwissProt protein database (https://www.uniprot.org/), applying an E-value threshold of 1×10^–5^. Only the top-scoring hit for each query sequence was retained.

Expression levels of the assembled genes and transcripts were calculated using RSEM v1.2.30 (Li and Dewey, 2011). Differential expression analysis and diagnostic plots were done using DESeq 2 (Love *et al.*, 2014) in R/Bioconductor. Only genes with an adjusted *P*-value <0.05 were considered as differentially expressed. Additional plots were generated using our own scripts in R, using the ggplot and ggrepel libraries. Analysis of overrepresented Gene Ontology (GO) terms was done separately for up- and down-regulated genes using clusterProfiler (Wu *et al.*, 2021). In each case, only the 1000 genes with the lowest adjusted *P*-value (as calculated with DESeq 2) and displaying at least a two-fold change in expression were considered.

### Statistical analysis

Statistical analysis was performed using Microsoft Excel and the PAST program. Student’s *t*-test was used for comparisons between two groups and one-way ANOVA was used for multiple group comparisons, followed by Tukey’s HSD test when significant differences were found.

## Results

### Formation of stress granules in lupin roots during hypoxia

**Fig. 1.**
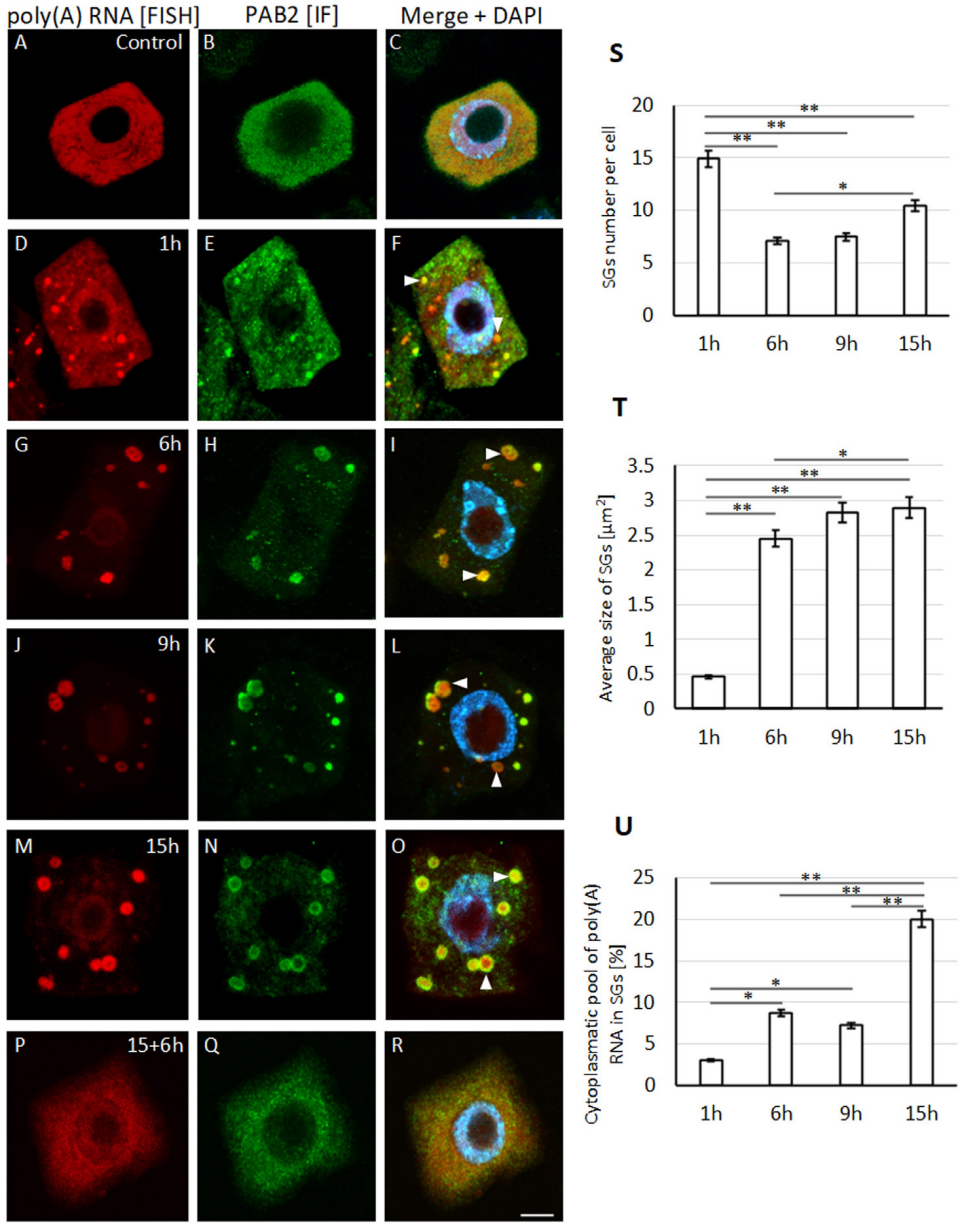
Localization of poly(A) RNA and stress granules in meristematic cells of lupin roots in response to hypoxia. Poly(A) RNA was detected by fluorescence *in situ* hybridization (FISH; red) and stress granules (SGs) were detected by immunofluorescence (IF; green) of Poly(A) Binding Protein 2 (PAB2). (A–C) Cells from roots under normoxia, (D–O) after 1–15 h of hypoxia, and (P–R) after 15 h of hypoxia followed by 6 h of reoxygenation. Merged images of the FISH and IF signals together with DAPI staining are shown. The scale bar is 10 µm. Arrowheads indicate the accumulation of poly(A) RNA during hypoxia; note their absence after the removal of stress. (S–U) Quantification of (S) the number of SGs per cell, (T) the size of SGs, and (U) the percentage of the cytoplasmatic pool of poly(A) RNA that was present in the SGs during the hypoxia stress. Significant differences between pairs of means were determined using Student’s *t*-test: *P<0.05, **P<0.01.

The localization of poly(A) RNA in root meristematic cells of lupin seedlings subjected to hypoxia and reoxygenation was examined (Fig. 1). In the cytoplasm, different-sized poly(A) RNA structures were observed during stress. To determine whether these structures could represent SGs, we performed localization of the SG-marker protein PAB2 (Fig. 1A–R). Strong co-localization of cytoplasmic aggregates, poly(A) RNA, and PAB2 was observed at all stages of hypoxia (Fig. 1D–O). Since the formation of SGs is inhibited when translation elongation is blocked, we treated roots during hypoxia with cycloheximide and found that the assembly of cytoplasmic aggregates was completely abolished in the meristematic cells of the treated samples (Supplementary Fig. S1). We therefore concluded that the cytoplasmic structures containing poly(A) RNA and PAB2 that formed in during hypoxia constituted SGs. Similar structures were also observed in non-meristematic root cells (Supplementary Fig. S2). Next, we examined SG formation during successive stages of hypoxia. After 1 h, round and similarly sized SGs appeared in the cytoplasm (Fig 1D–F). At 6 h, the area of each individual SG increased significantly, but the number of SGs per cell decreased (Fig. 1S, T). They appeared elongated and to be composed of two or more circular structures (Fig. 1G–I), strongly suggesting the fusion of single SGs into larger structures. The fusion of SGs continued in the cells of roots exposed to hypoxia stress for 9 h and 15 h (Fig. 1J-O) and the number of SGs slightly increased (Fig. 1S). When the stress was removed after 15 h and the seedlings were cultured for 6 h in an optimal oxygen concentration (reoxygenation), the SGs were no longer observed (Fig. 1P–R). After the first hour of stress, a high level of poly(A) RNA was observed in the cytoplasm of the root cells, accompanied by the appearance of small stress granules that contained 3.3% of the cytoplasmic transcripts (fluorescence signal per SG; Fig. 1U); however, in cells stressed for 6 h and 9 h, the quantity of poly(A) RNA increased in the SGs. Hypoxia for 15 h resulted in the highest poly(A) RNA accumulation in SGs, accounting for 20.4% of the total cytoplasmic pool. Our results suggested that the stages of SG formation consisted of the appearance of small structures, which then merged into larger ones, leading to a decrease in their number. In addition, we also showed that poly(A) RNA accumulation in the SGs increased proportionally with the duration of the stress while its level in the cytoplasm decreased (Fig. 1D–O), indicating that the poly(A) RNA in the SGs originated in the cytoplasm.

### Bi-zonal stress granules are present in the roots of lupin

**Fig. 2.**
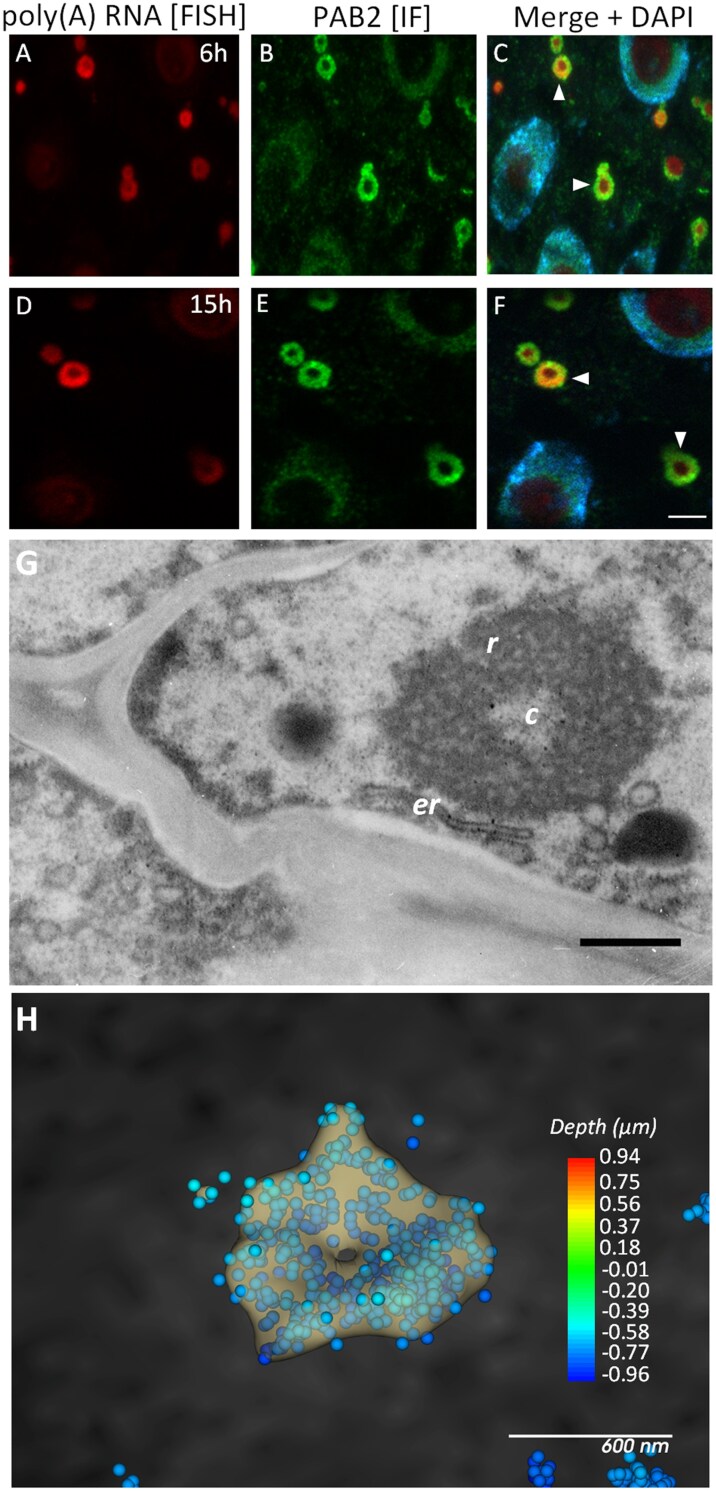
Localization of poly(A) RNA and stress granules in resin sections of meristematic cells of lupin roots in response to hypoxia. Poly(A) RNA was detected by fluorescence *in situ* hybridization (FISH; red) and stress granules (SGs) were detected by immunofluorescence (IF; green) of Poly(A) Binding Protein 2 (PAB2). Cells after (A–C) 6 h and (D–F) 15 h of hypoxia. Merged images of the FISH and IF signals together with DAPI staining are shown. Arrowheads indicate SGs. The scale bar is 5 µm. (G) Ultrastructure of the bi-zonal form of the SGs; *r*, ring of coiled, dense fibers; *c*, central brighter area; *er*, endoplasmatic reticulum. Scale bar is 3 µm. (H) Stochastic optical reconstruction microscopy (STORM) analysis. The dots represent individual poly(A) RNA molecules that were detected during the acquisition and are color-coded by depth. The dot size in the image is set to 50 nm. The SG structure in the image is the isosurface of the cluster calculated by performing a Cluster analysis on the data set.

We observed that PAB2 and poly(A) RNA were not homogeneously distributed within the SGs. To confirm this observation, FISH and immunochemical reactions on resin sections were performed. Both poly(A) RNA and PAB2 were found at the periphery of the SGs, forming a ring with space in the center that was free of the two compounds. These types of SGs were primarily observed after 6–15 h of hypoxia (Fig. 2A–F). PAB2 formed fine clusters, while poly(A) RNA had a more uniform distribution in the ring. To further verify this observation, the SGs were next examined by TEM. The SGs found in hypoxic roots exhibit a two-zone structure, consisting of a ring formed of coiled, dense fibers and a central area that appeared brighter (Fig. 2G). SGs presenting this morphology have not previously been described in plants. SGs were not observed at the EM level under normoxic conditions (Supplementary Fig. S3A). Immunogold labeling confirmed the presence of PAB2 in SGs (Supplementary Fig. S3C). Next, we used STORM with a high-resolution confocal microscope to examine the distribution of poly(A) RNA. In Fig. 2H, the dots represent the individual localizations of molecules detected during the acquisition, and it shows that the signal was uniformly distributed over the ring of the SGs but it did not occur in the center of this structure, confirming the two-zone nature of the SGs. We also observed similar bi-zonal morphology of SGs in non-fixed material after isolating SGs from lupin root cells (Supplementary Fig. S4). Curiously, we observed that the SGs were often bordered by endoplasmic reticulum (Fig. 2G). In the central zone there were ribosomes with the same structure as those as in the cytoplasm, including the ones on the endoplasmic reticulum (Fig. 2G; Supplementary Fig. S3B). To confirm the ribosomes in the central zone, we examined the localization of *18S* and *26S* rRNAs (i.e. the small and large subunits; Supplementary Fig. S5A–L), and observed both in the center of the SGs at levels similar to those in the cytoplasm (Supplementary Fig. S5M, N). The detected levels of both *26S* and *18S* rRNA were significantly lower in the ring (fluorescence signal of rRNA per µm^2^). On the other hand, only *26S* rRNA showed a higher accumulation around the SGs (Supplementary Fig. S5D–F). Taken together, our results provide the first evidence to prove the presence of heterogeneous SGs in plants. Moreover, we also show that the central region of these SGs contains ribosomes at levels similar to those of cytoplasm.

### RNA-seq reveals that the transcriptome of lupin is significantly affected by hypoxia

**Fig. 3.**
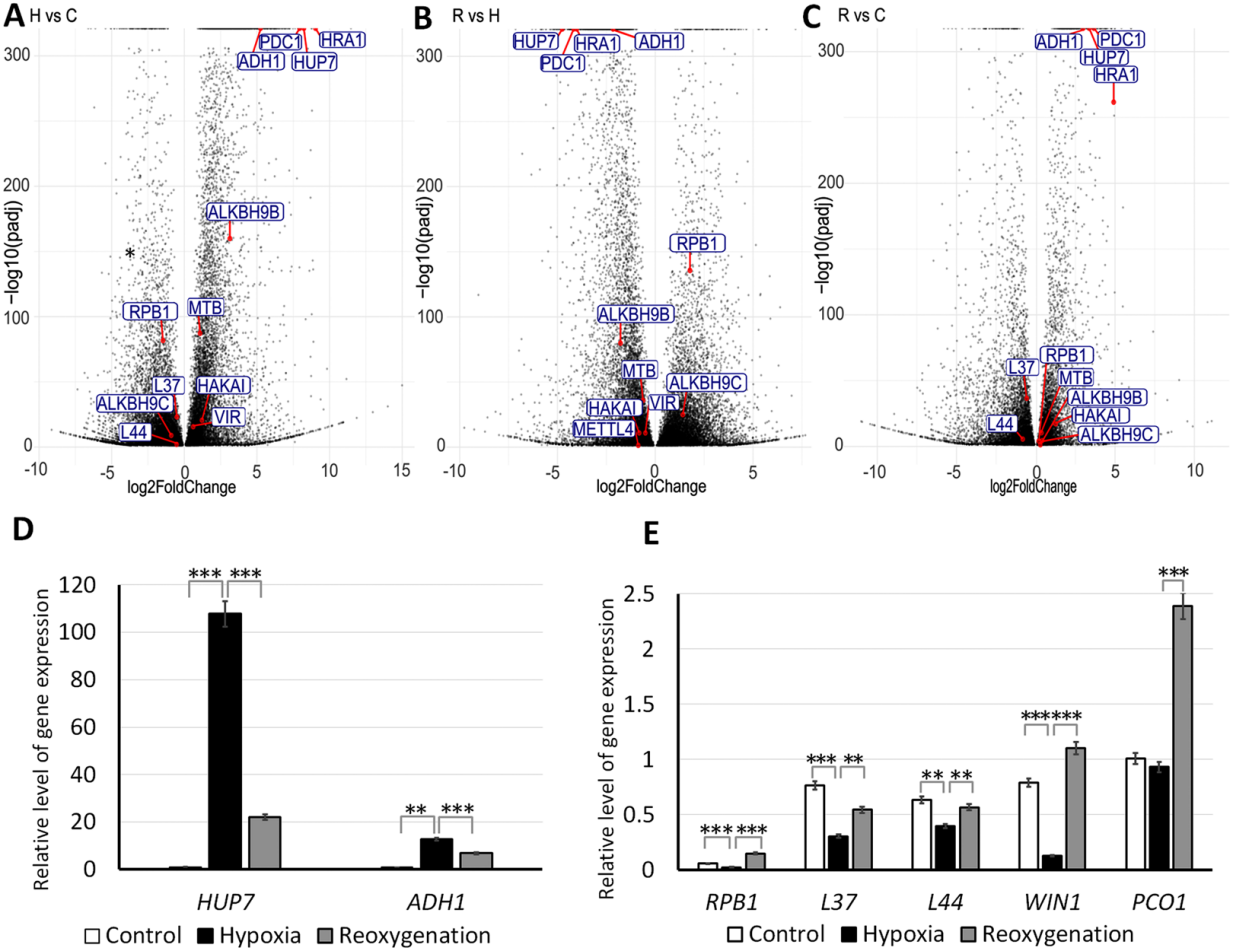
Differential expressed genes (DEGs) in lupin roots in response to hypoxia and subsequent reoxygenation. Seedlings were submerged for 15 h (hypoxia, H) after which the stress was removed for 6 h (reoxygenation, R); no stress was applied to control seedlings (normoxia, C). (A–C) Volcano plots of DEGs identified in comparisons of (A) hypoxia versus normoxia, (B) reoxygenation versus hypoxia, and (C) reoxygenation versus normoxia. Selected genes are labeled. Hypoxia-responsive genes: *Alcohol Dehydrogenase 1* (*ADH1*); *Hypoxia-response Unknown Protein 7* (*HUP7*); *Pyruvate Decarboxylase 1* (*PDC1*); and *Hypoxia Response Attenuator 1* (*HRA1*). m^6^A writer genes: *Methyltransferase B* (*MTB*); *Virilizer* (*Vir*); and the E3 ubiquitin-protein ligase *HAKAI*. m^6^A eraser genes: demethylases *ALKBH9B* and *ALKBH9C*. Housekeeping genes: 60S ribosomal proteins L37 (*L37*) and L44 (*L44*); and the DNA-directed RNA polymerase II subunit (*RPB1*). (D, E) qRT-PCR quantification of expression levels of genes identified as (D) hypoxia markers genes and (E) housekeeping genes under normoxia, hypoxia, and reoxygenation. Expression levels are relative to that of the reference gene *UBC5*. Data are means (±SE) of three independent biological replicates. Significant differences among means were determined using Student’s *t*-test: **P<0.01, ***P<0.001.

To identify differentially expressed poly(A) RNAs in response to hypoxia, we conducted high-throughput sequencing of lupin roots that had been exposed to three different treatments, namely normoxia, hypoxia, and hypoxia followed by reoxygenation, and performed an *ab initio* transcriptome assembly (Supplementary Dataset S1; Supplementary Table S2). To examine potential systematic patterns across biological replicates, we performed principal component analysis (PCA; Supplementary Fig. S6A). Hierarchical clustering revealed limited variation among the three biological replicates, and very clear distinction between the three treatments, confirming the remarkably strong response of the plants to the different oxygen conditions (Supplementary Fig. S6B). In all cases, we observed similar numbers of genes to be up- and down-regulated (Fig. 3A–C; Supplementary Table S3). The largest number of differentially expressed genes (DEGs) were found between hypoxia and the normoxia control (21 036), confirming the robust response to hypoxia. Importantly, the DEGs in this group contained several hypoxia-responsive genes (HRGs) that have previously been identified in Arabidopsis (Mustroph *et al.*, 2010). These HRGs, namely *Alcohol Dehydrogenase 1* (*ADH1*), *Hypoxia-response Unknown Protein 7* (*HUP7*), *Pyruvate Decarboxylase-1* (*PDC1*), and *Hypoxia Response Attenuator1* (*HRA1*), were up-regulated in hypoxia, with fold-changes ranging between 5.8 and 8.9 (Fig. 3A). Interestingly, among the other up-regulated DEGs, we also observed genes related to m^6^A-metabolism, such as the m^6^A-writers *Methyltransferase B* (*MTB*), *Virilizer* (*Vir*), and the E3 ubiquitin-protein ligase *HAKAI* (Fig. 3A). However, not all the genes linked to m^6^A followed the same trend; for instance, the m^6^A-demethylase (eraser) *ALKBH9B* was up-regulated whilst its close homolog *ALKBH9C* displayed decreased expression levels under stress. Notably, housekeeping genes showed reduced expression during stress, as demonstrated by those encoding the 60S ribosomal protein L44 (*L44*), the 60S ribosomal protein L37 (*L37*), and the DNA-directed RNA polymerase II subunit (*RPB1*).

As expected, when comparing reoxygenation to hypoxia, the patterns described above were reversed (Fig. 3B). We also examined the transcriptomic changes after reoxygenation compared with normoxia. Intriguingly, the HRG genes remain active after the removal of stress but to a lesser extent than during the hypoxia, with fold-changes ranging between 2.5. and 5 (Fig. 3C). In this set of DEGs, we found that both m^6^A-writers and -erasers were up-regulated compared to normoxia. Meanwhile, expression of the *RPB1* housekeeping gene was up-regulated but that of *L37* and *L44* was not.

To comprehensively explore the regulatory networks involved in the hypoxia response, we first conducted a GO analysis on the sets of up- and down-regulated genes during stress compared to normoxia. The up-regulated DEGs were categorized into 24 GO terms (Supplementary Fig. S7A). The primary six sub-networks were protein serine/threonine kinase activity, regulation of cell wall including xyloglucan, trehalose biosynthetic process, sucrose metabolism, ABA response, and oxidoreductase activity. Among the down-regulated genes, several categories were associated with various cellular functions such as organelle function, cytoskeleton, and vesicle transport, specifically heme binding, proteolysis, transmembrane transporter activity and cellular oxidant detoxification (Supplementary Fig. S7B). We then conducted a GO analysis on the up- and down-regulated genes for reoxygenation versus hypoxia (Supplementary Fig. S7C, D). The up-regulated genes were classified in 25 categories, including genes involved in heme binding, transmembrane transporter activity, metabolic process, and structural constituent of the cytoskeleton (Supplementary Fig. S7C). Genes with decreased transcriptional activity were protein serine/threonine kinase activity, regulation of cell wall including xyloglucan, trehalose biosynthetic process, and sucrose metabolism(Supplementary Fig. S7D). The GO analysis of reoxygenation versus hypoxia revealed several contrasting trends compared to hypoxia versus normoxia (Supplementary Fig. S7E, F), indicating a robust recovery after the stress period. Notably, all the comparisons underscored strong regulation of biogenesis and cell wall components.

We selected several genes that were shown to be up- or down-regulated by the RNA-seq data for validation by qRT-PCR. Of the HRGs known from Arabidopsis, we examined *ADH1*, *HUP7*, *Plant Cysteine Oxidase 1* (*PCO1*), and the ethylene-responsive transcription factor (*WIN1*). For the first two, a significant increase of transcripts was observed during hypoxia, consistent with the RNA-seq results, and subsequent reoxygenation resulted in a reduction in the amount of mRNAs (Fig. 3D). In contrast, during stress the expression of *PCO1* did not change whilst that of *WIN1* strongly decreased (Fig. 3E), indicating that these homologs do not constitute HRGs in lupin. Meanwhile, the transcriptional activity of the housekeeping genes *L37*, *L44*, and *RPB1* decreased during hypoxia and increased after stress removal, confirming that they were not responsive to oxygen reduction in lupin. Thus, the qPCR results strongly confirmed the RNA-seq data.

### Differential localization of mRNAs in stress granules during hypoxic stress

**Fig. 4.**
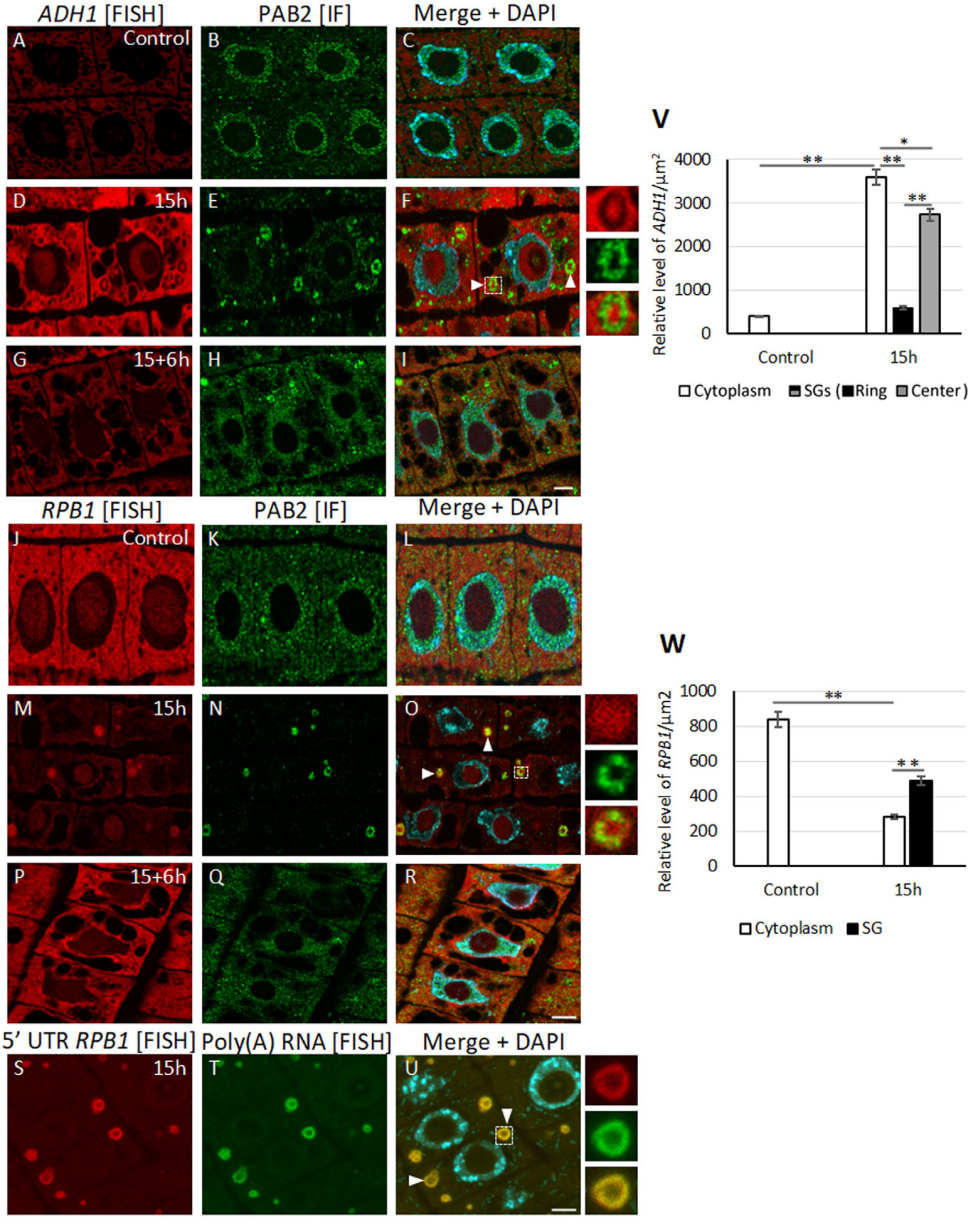
Localization of *ADH1* and *RPB1* mRNA and stress granules in meristematic cells of lupin roots in response to hypoxia and subsequent reoxygenation. Seedlings were submerged for 15 h (hypoxia; ‘15h’) after which the stress was removed for 6 h (reoxygenation; ‘15+6h’); no stress was applied to control seedlings (normoxia). Poly(A) RNA was detected by fluorescence *in situ* hybridization (FISH; red) and stress granules (SGs) were detected by immunofluorescence (IF; green) of Poly(A) Binding Protein 2 (PAB2). Merged images of the signals with DAPI staining are shown. (A–I) *Alcohol Dehydrogenase 1* (*ADH1*), (J–R) the DNA-directed RNA polymerase II subunit (*RPB1*), and (S–U) the 5´-UTR of *RPB1*. Arrowheads indicate SGs. Scale bars are 10 µm. The panels to the right show magnified images of the SG that is marked with a square. Homogenous distribution of *RPB1* transcripts in all areas of the SGs was detected by FISH using a probe targeting the middle sequence of mRNA (arrowheads in O), whereas when a probe targeting the 5´-UTR of *RPB1* was used transcripts were only visible in the ring of the SGs (arrowheads in U). *ADH1* transcripts were present only in the central zone of the SGs (arrowheads in F). (V, W) The relative fluorescence intensities of mRNA of (V) *ADH1* and (W) *RPB1* in the cytoplasm and SGs during normoxia and hypoxia. Data are means (±SE) of three independent biological replicates. Significant differences between means were determined using Student’s *t*-test: *P<0.05, **P<0.01.

To understand the spatial relationship between selected mRNAs and SGs, we examined the localization and quantity of transcripts (measured as fluorescence signal per µm^2^) with different expression levels within the cells. The level of *ADH1* mRNA significantly increased under hypoxic stress (Fig. 4A–F). Within the SGs, three different probes for *ADH1* revealed a non-uniform distribution, with the transcript predominantly localized in the central region of the granules, while being almost absent in the surrounding ring-like region (Fig. 4D–F). Quantitative measurements of *ADH1* mRNA in the different SGs regions and the cytoplasm showed that the ring-like zone contained over four times less mRNA compared to the central zone and cytoplasm (Fig. 4V). The low amount of *ADH1* in the ring-like zone was comparable to the mRNA levels in the cytoplasm under control conditions. In contrast, the amount of transcripts in the central part of the SGs was only about 15% lower than in the cytoplasm of cells exposed to 15 h of stress. The distribution of *ADH1* in the SGs and the cytoplasm was similar to that of rRNA (Supplementary Fig. S5D–F, J–L). During reoxygenation, the SGs disappeared, and the amount of *ADH1* transcript in the cytoplasm decreased (Fig. 4G–I).

We next examined the localization of transcripts of housekeeping genes. FISH using probes targeting the central region of the mRNA sequence confirmed the qPCR and RNA-seq results for *RPB1* (Fig. 4J–R), demonstrating that transcript levels decreased during hypoxia and strongly increased after stress removal. Hypoxic stress led to a decrease in transcript levels in the cytoplasm and an increase in SGs (Fig. 4M–O). In contrast to *ADH1*, the distribution of *RPB1* transcripts within the SGs appeared relatively uniform. The level of *RPB1* mRNA in SGs was nearly twice as high as in the cytoplasm following prolonged hypoxic stress (15 h; Fig. 4W). This suggested that the decrease in transcript levels in the cytoplasm correlated with their accumulation in the SGs. Upon reoxygenation, the dispersal of these structures was accompanied by a strong signal in the cytoplasm (Fig. 4P–R). A similar localization pattern was observed for other transcripts, including the hypoxia-response gene *HUP7* and the four housekeeping genes *L37*, *L44*, *PCO1*, and *WIN1* (Supplementary Figs S8, 9). The localization of the various mRNAs revealed their diverse distribution within SGs. The presence and localization within SGs depended on the amount of mRNA and the function of the protein encoded by it.

In animal cells, it has been shown that both translatable and untranslatable mRNAs are present in SGs, and that translation within SGs is not rare (Mateju *et al.*, 2020). One of the mechanisms of translation regulation might be the availability of different sequences of mRNA to ribosomes. To determine whether the localization of RNA within the SGs regulated translation, the distribution of the 5´-UTR of *RPB1* was examined (Fig. 4S–U). Interestingly, a slightly different distribution was obtained compared with the probe recognizing the middle of the *RPB1* mRNA sequence. The 5´-UTR of *RPB1* was exclusively found in the ring of the SGs, similar to the probe to poly(A) RNA. This suggests distinct positioning of the 5´-end, 3´ends, and the middle sequences of the transcripts. The mRNA ends were located in the ring of the SGs, where there was no rRNA, while the middle of the transcripts was observed in the central area of the SGs. This implies that the 5´-end of mRNA is not accessible for initiation of protein translation.

### Localization and levels of m^6^A in the lupin root cells

Recent studies have demonstrated the presence of the m^6^A-binding protein ECT2 in SGs of Arabidopsis (Kosmacz *et al.*, 2019; Fan *et al.*, 2023), and hence we aimed to investigate whether RNAs with m^6^A were present in the SGs of lupin. First, the specificity of the antibodies was assessed. No signal or a very strong reduction in signal was observed after omitting the primary antibody, in sections previously treated with RNase, and also in the Arabidopsis *pABI3::MTA-mta* mutant line with lower amounts of m^6^A in RNA (Supplementary Fig. S10).

**Fig. 5.**
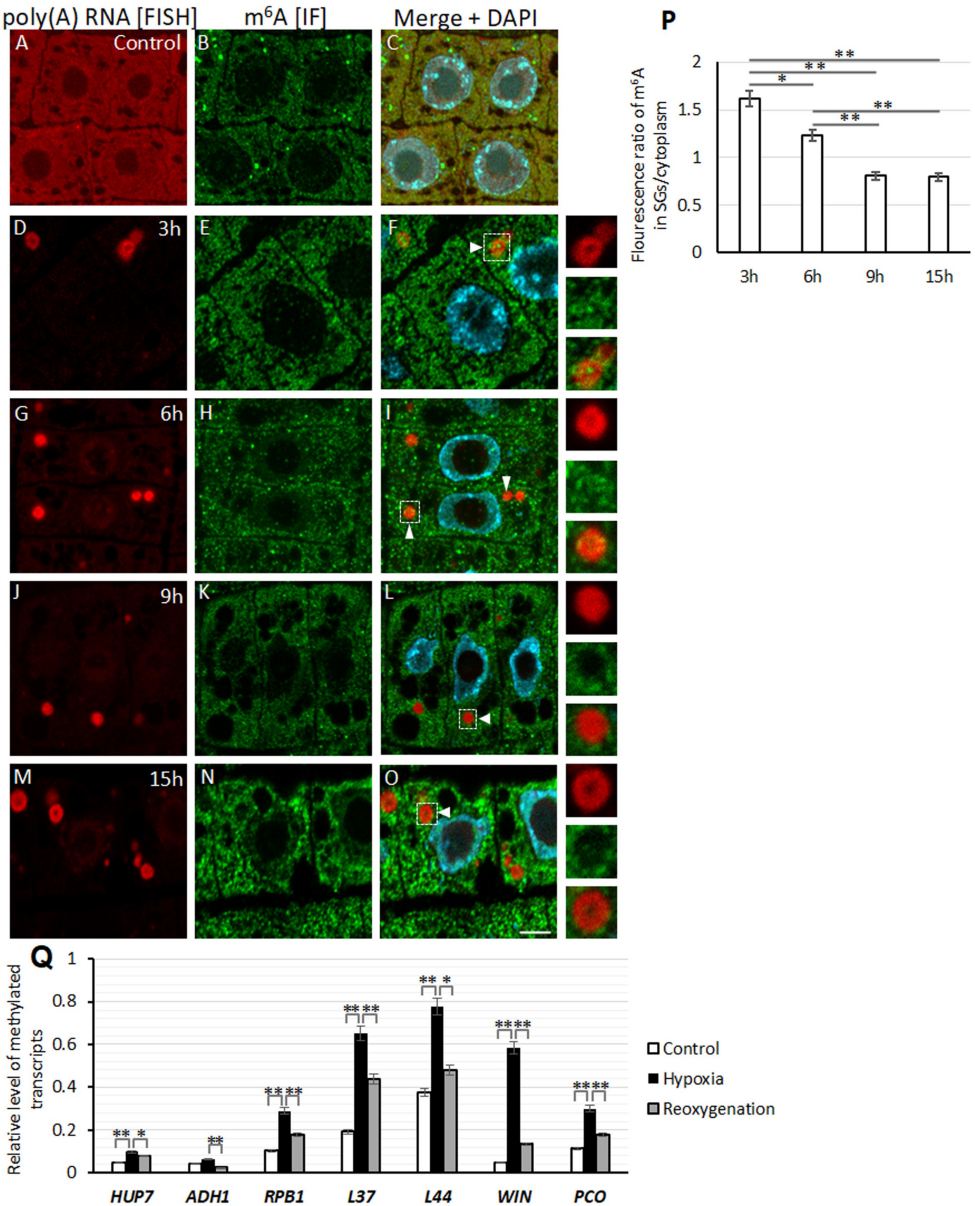
Localization of poly(A) RNA and m^6^A in meristematic cells of lupin roots in response to hypoxia. Poly(A) RNA was detected by fluorescence *in situ* hybridization (FISH; red) and m^6^A was detected by immunofluorescence (IF; green). Merged images of the signals with DAPI staining are shown. (A–C) Normoxia conditions (Control) and (D–O) hypoxia conditions for 3–15 h. The panels to the right show magnified images of the stress granule (SG) that is marked with a square. The scale bar is 10 µm. Accumulation of m^6^A in SGs was observed during 3–6 h of hypoxia (arrowheads in F, I) followed by a reduction during 9–15 h (arrowheads in L, O). (P) Ratio of fluorescence signal in SGs compared with the cytoplasm during 3–15 h of hypoxia. (Q) Quantitative measurements of methylation (m^6^A modification) in transcripts of *HUP1*, *ADH1*, *RPB1*, *L37*, *L44*, *WIN1*, and *PCO1* in lupin roots subjected to normoxia (control), hypoxia for 15 h, and hypoxia followed by reoxygenation for 6 h. Gene names are listed in full in (Fig. 3). Data are means (±SE) of three independent biological replicates. Significant differences between means were determined using Student’s *t*-test: *P<0.05, **P<0.01.

In lupin roots under control conditions, the poly(A) RNA and m^6^A signals strongly overlapped (Fig. 5A–C). Dynamic changes in the level of m^6^A in the SGs were observed in during the course of hypoxia (Fig. 5D–O). During the first hours of stress (3–6 h), m^6^A accumulated in SGs (Fig 5D–I), whilst a reduction in m^6^A within the SGs was observed thereafter (Fig. 5J–O). The ratio of m^6^A in the SGs relative to the cytoplasm was highest at the onset of stress and had decreased by 50% after 9–15 h of hypoxia (Fig. 5P), indicating accumulation of m^6^A-rich RNA in the early stages of SGs assembly followed by a reduction during stress. To investigate the relationships between the level of m^6^A and the localization and expression levels of transcripts, we employed the MeRIP technique. The results indicate substantial variations in m^6^A levels among mRNAs (Fig. 5Q). Except for *ADH1*, there were significant increases in m^6^A levels during hypoxia, and all transcripts showed a decrease during reoxygenation. *ADH1* and *HUP7* mRNAs, which had the highest expression levels in hypoxia, exhibited significantly lower quantities of m^6^A at all stages compared to the other genes. It is noteworthy that a higher increase in m^6^A levels during hypoxia compared to normoxia correlated with the decreased amount of mRNA among the housekeeping genes (Fig. 3E). This relationship was particularly evident for *WIN1* and *L37*. These results indicated a clear negative correlation between the m^6^A level and the expression of the studied transcripts, and this was observed across all the genes examined and all the stages. On the other hand, despite its equally low level of m^6^A, *HUP7* was present in both the SGs and the cytoplasm. Notably, the expression of *HUP7* was the highest among all the mRNAs analysed during hypoxia, surpassing even *ADH1*. These results confirmed those of the NGS, which showed 1588 and 9635 reads for *ADH1* and *HUP7*, respectively, in hypoxia, compared to 432 and 9 reads, respectively, in normoxia. Given this, it can be concluded that the m^6^A modification level in RNA affects the quantity and localization of transcripts in the SGs.

### Impact of m^6^A on the formation and accumulation of poly(A) RNA in SGs in Arabidopsis

**Fig. 6.**
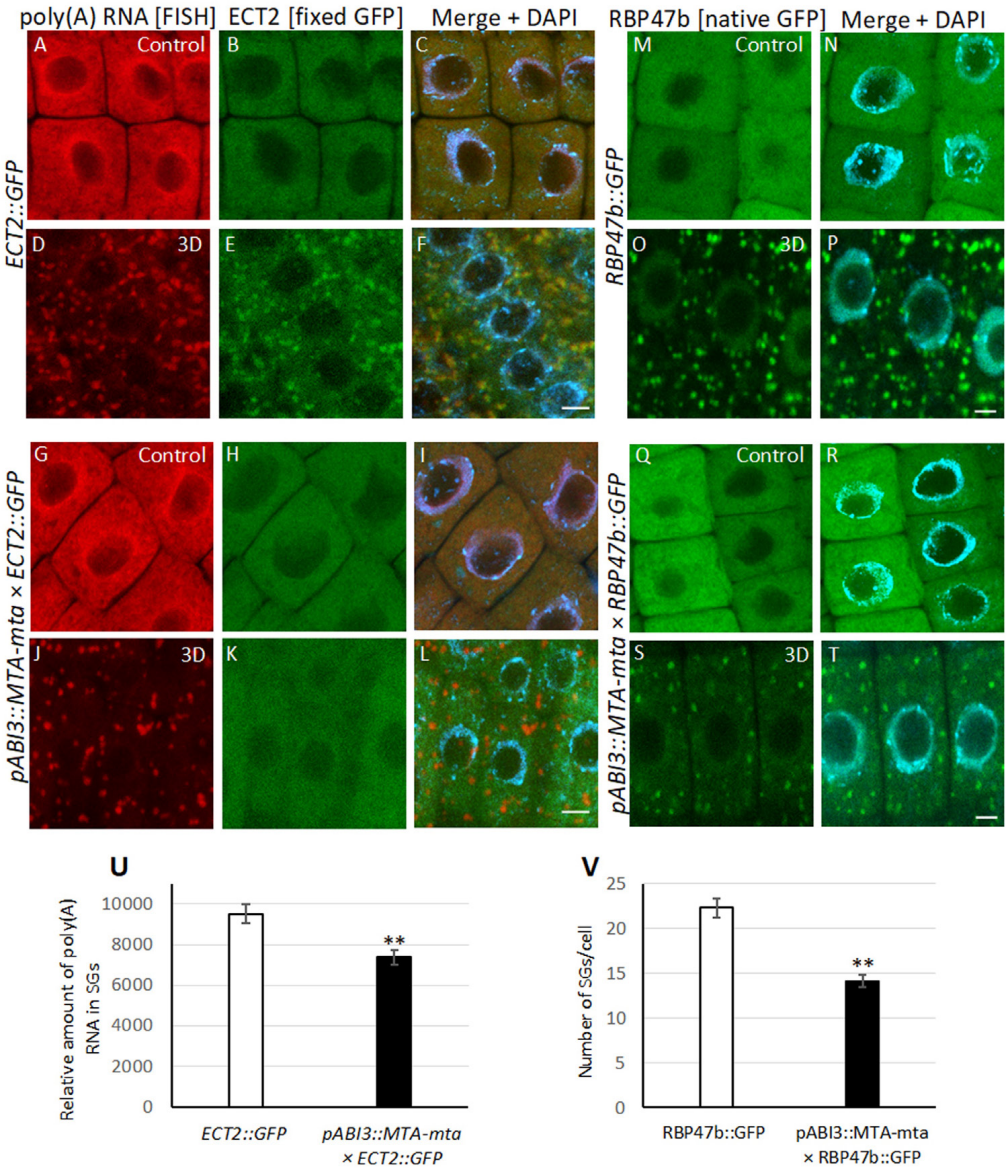
Localization of poly(A) RNA, the m^6^A-binding protein ECT2, and the DNA-directed RNA polymerase protein RBP47b in Arabidopsis roots cells in response to long-term hypoxia. Seedlings were subjected to normoxia (Control) conditions or 3 d of hypoxia (3D). Poly(A) RNA was detected by fluorescence *in situ* hybridization (FISH; red), and ECT2 and RBP47b were detected by fluorescence of GFP (green). Merged images of the signals with DAPI staining are shown. (A–F) The *ECT2::GFP* line and (G–L) the *mta* mutant transformed with the *pABI3::MTA* construct (*pABI3::MTA-mta*) crossed with the *ECT2::GFP* line. (M–P) The *RBP47b::GFP* line and (Q–T) the *pABI3::MTA-mta* line crossed with the *RBP47b::GFP* line. Scale bars are 10 µm. (U) The relative level of poly(A) RNA in the stress granules (SGs) of the *ECT2::GFP* line and the *pABI3::MTA-mta* × *ECT2::GFP* cross under hypoxia. (V) The number of SGs per cell in the *RBP47b::GFP* line and the *pABI3::MTA-mta* × *RBP47b::GFP* cross under hypoxia. Data are means (±SE) of three independent biological replicates. Significant differences were determined using Student’s *t*-test: **P< 0.01).

Mutants of *L. angustifolius* are not available, and therefore in order to study the function of m^6^A in the formation of SGs we used the *pABI3::MTA*-*mta* Arabidopsis mutant line. The *ABI3* promoter drives strong embryo expression of *MTA*, resulting in a high level of m^6^A that bypasses embryo lethality. However, this promoter also drives a very low level of expression post-germination (in 14-day-old seedlings), giving rise to plants with 80–90% lower amounts of m^6^A in RNA than their wild-type counterparts (Bodi *et al.,* 2012). We crossed the *pABI3::MTA*-*mta* lines with the *ECT2::GFP* (a reader of m^6^A occurring in SGs) and *RBP7b::GFP* (a marker of SGs) lines, and microscopic analysis showed a lack of accumulation of ECT2 in the cytoplasm of plants with decreased levels of m^6^A (Fig. 6A–L). Interestingly, poly(A) RNA clusters resembling SGs were observed in the cytoplasm. This shows that only ECT2 with m^6^A RNA can accumulate in SGs, and ECT2 is not a crucial protein for the assembly of SGs. Next, we studied the impact of m^6^A on the quantity of SGs in cells in the *pABI3:MTA-mta* × *RBP47b::GFP* lines (Fig. 6M–T) and found that the number was ~35% lower than in the *RBP47b::GFP* control (Fig. 6V). The potential involvement of m^6^A in the formation of SGs was evidenced by a slightly lower level of poly(A) RNA (relative quantity per SG) in the *pmtaABI3:MTA* × *ECT2::GFP* lines with decreased levels of m^6^A (Fig. 6U). Similar to lupin, the m^6^A level in SGs of Arabidopsis during long-term hypoxia was relatively low compared to the cytoplasm (Supplementary Fig. S11). These results suggest that m^6^A might play a role in the initial stages of formation of SGs and in the quantity of mRNA that they contain.

## Discussion

Hypoxia stress leads to changes at the morphological, physiological, and molecular levels. To better understand the mechanisms of the stress response, we conducted a study of transcriptomic changes and an examination of SG formation in lupin seedlings subjected to submergence. The high specificity of the response to submergence at the transcriptome level was evidenced by a strong correlation between genes that were highly expressed during stress and those that decreased in expression after stress removal (Fig. 3A, B). GO analysis of the transcriptomic data revealed that in response to submergence in lupin roots, genes associated primarily with serine/threonine kinase activity, central carbon metabolism, and cell wall biogenesis were highly expressed (Supplementary Fig. S7). Protein kinases play a key role in plant responses to stress through sensing and signal transduction (Chen *et al.*, 2021). In turn, the maintenance of cell wall functional integrity and physical properties through the modification of their components is an important process during responses to both biotic and abiotic stresses (Le Gall *et al.* 2015; Vaahtera *et al.*, 2019; Rui and Dinneny, 2020; Baez *et al.*, 2022). Our transcriptome data demonstrated that the rapid changes in cell wall architecture during submergence are crucial in the lupin stress response and adaptation. Most of the mRNAs that showed increases in expression were associated with the loosening or remodeling of the cell wall. It is known that modification of root anatomy during waterlogging enhances oxygen availability to cells and reduces oxygen loss from the roots to the soil (Bramley *et al.*, 2011). The results of our RNA-seq indicated that hypoxia was the primary component of submergence stress, and that one of the key response mechanisms in lupin roots is the remodeling of the cell wall (Fig. 3A; Supplementary Fig. S7), which facilitates oxygen retention and intercellular transport.

Serine/threonine kinases also play a crucial role in the adaptive mechanisms of lupin during submergence (Chen *et al.*, 2021). One of the highly expressed kinase genes that we identified was *Casein Kinase 2* (*CK2*; TanjilG_11075; Supplementary Table S3), which encodes a pleiotropic enzyme that plays a major role in abiotic stress responses and regulates plant growth (Agarwal and Ray, 2020). Interestingly, CK2 is also closely related to SG dynamics and the regulation of the translation process: under stress conditions it accumulates in SGs, and its phosphorylation of the nucleating protein G3BP1 leads to the disassembly of SGs in mammalian cells when stress conditions subside (Reineke *et al.*, 2017). In plants, CK2 also phosphorylates the translation initiation factor eIF5A, which has been shown to play a role in SG assembly in animals cells (Li *et al.*, 2010; Muench and Dahodwala, 2012). Our results showed a strong increase in the level of *CK2* mRNA in hypoxia in lupin, followed by a decrease after reoxygenation (TanjilG_11075; Supplementary Table S3). This suggests the involvement of CK2 in the assembly rather than in the breakdown of SGs in plants; however, further research is needed.

We made a detailed examination of the emergence of SGs in the cells of lupin roots during hypoxia stress. These structures were formed by the nucleation of poly(A) RNA and PAB2, which then fused into larger SGs (Fig. 1). This is consistent with previous studies on the number and size of SGs in Arabidopsis, where prolonged heat stress causes a decrease in the number and a slight increase in the volume of the structures (Gutierrez-Beltran *et al.*, 2015; Hamada *et al.*, 2018). However, we found differences in the diameter and shape compared with Arabidopsis. In lupin, the SGs were a few times larger than in Arabidopsis and consisted of two zones: a ring and a central area (Figs 1, 2), which differed in composition and function. The ring consisted of coiled fibers rich in poly(A) RNA, PAB2, and transcripts of all the genes that we examined except *ADH1* (Fig. 4; Supplementary Figs S8, S9). The distribution of molecules in the ring-like part was homogeneous. Analysis of poly(A) RNA under a high-resolution microscope did not reveal any clusters that could resemble the cores of SGs. On the other hand, the central part of the SGs lacked the poly(A) RNA tail and the PAB2 protein (Fig. 2). In addition, analysis of various mRNA fragments revealed that in the ring of SGs there were 5´-UTR *RPB1* mRNA and 3´-poly(A) mRNAs, while the middle sequences of *L37*, *L44*, and *RPB1* were present in both parts of SGs. This indicates that anchoring the 5´- and 3´-ends of mRNA in the SG ring might inhibit the association of the small and large subunits of the ribosome to transcripts. This suggestion is supported by the localization of the entire *ADH1* mRNA sequence exclusively in the central region (Fig. 4), together with *18S* and *26S* rRNA and ribosomes (Supplementary Fig. S5), which are necessary for its translation. *ADH1* is believed to be essential for surviving hypoxic stress (Dennis *et al.*, 2000). In animal cells, it has been shown that both translatable and untranslatable mRNAs are present in SGs, but the mechanism that regulates this process remains unknown (Mateju *et al.*, 2020). According to our results, SGs in lupin are much larger and exhibit a bi-zonal structure, and the regulation of synthesis proteins might be related to the different distributions of the 5´- 3´-ends and middle sequence of the transcripts within the SGs.

We studied the possibility of m^6^A participation in SG assembly in lupin under hypoxia. In animal cells, N^6^-methyladenosine has been proposed to target transcripts to SGs (Fu and Zhuang, 2020; Ries *et al.*, 2023), and these modifications can affect the propensity of mRNA to form LLPS aggregates or to interact with SG-associated proteins. It has been shown in oxidative stress that levels of m^6^A modification significantly increase in human cell lines, mainly in the 5´-UTR and 5´-region of the coding sequences of SG-associated transcripts (Anders *et al.*, 2018). In the Arabidopsis model, Kosmacz *et al.* (2019) have shown the presence of the well-characterized m^6^A readers ECT2 and ECT4 in SGs under heat stress. It has also been shown that ECT2 (YTH9) is re-localized from the cytoplasm to the SGs under heat shock (Scutenaire *et al.*, 2018). Our results indicated the presence of m^6^A-modified RNA in SGs, as we observed that ECT2 did not accumulate in SGs in an Arabidopsis mutant with reduced m^6^A levels (Fig. 6). In lupin, the mRNAs of housekeeping genes with high levels of m^6^A (determined by MeRIP using whole cells) (Fig. 5) were mostly present in the SGs during hypoxia stress (Fig. 4), suggesting the involvement of m^6^A in the accumulation of transcripts in SGs. The exception was *HUP7* where, despite the low level of m^6^A, its mRNAs were found in SGs; however, this gene had the highest expression level among all the mRNAs studied (Fig. 3). It has been shown that regardless of their function, RNAs can be accumulated in SGs (Tong *et al.* 2022). This might depend on the amount of mRNA, as observed in ER-stress in Arabidopsis where the decline in translation efficiency results in transcripts of the unfolded protein response (UPR) not being loaded onto polyribosomes in response to the stress; instead, the mRNA accumulates in SGs (Kanodia *et al.*, 2020). It has been observed across different models that upon polysome dissociation, free exogenous mRNA delivery creates a high local RNA concentration that triggers SG assembly (Campos-Melo *et al.*, 2021). Our results for *HUP7* indicate that high transcriptional activity might also contribute to the local increase of mRNA in the cytoplasm and result in recruitment to the SGs. Interestingly, *ADH1* mRNAs with low levels of m^6^A and lower expression than *HUP7* were located outside of the ring of SGs in lupin (Fig. 4). On the other hand, as a result of its high expression level, *HUP7* mRNAs depleted of m^6^A were present in both the SGs and the cytoplasm (Supplementary Fig. S8). This therefore indicates that the balance between levels of m^6^A and transcripts was responsible for the localization of mRNAs in SGs.

The amount of m^6^A was relatively high at the beginning of stress, but the quantity of modified nucleotides in SGs in lupin decreased with time as the stress conditions persisted (Fig. 5). We also did not observe a strong accumulation of m^6^A in SGs in Arabidopsis under long-term hypoxia (Supplementary Fig. S11). However, in mutants we found that lowering the level of m^6^A in the *pABI3::MTA-mta* line blocked ECT2 accumulation and reduced the number of SGs, and this was also accompanied by a slight reduction in the amount of poly(A) RNA in the SGs (Fig. 6). We have demonstrated in lupin that an increase in quantity of transcripts and the level of m^6^A modification regulates their localization within SGs. This indicates the involvement of m^6^A in the assembly and composition of the SG transcriptome in plants, especially in the first steps of their formation. Nevertheless, RNA recruitment to SGs appears to be a multilayered regulatory process in plants. In animals, while most studies highlight the key role of m^6^A in mRNA recruitment to SGs, it has also been shown that mRNA length and its cytoplasmic concentration are important factors in this process (Sun *et al.*, 2023).

The decrease in the amount of m^6^A in SGs that we observed during hypoxia stress (Fig. 5) requires further research, although recent results from Fan *et al.* (2023) have revealed a possible mechanism. They showed that the *Onsen* RNA transposon with m^6^A methylation is transported to SGs containing the demethylase ALKBH9B in biotic stress. In the *alkbh9b* mutant, an increase in transposon levels was observed in seedlings, including in SGs. The results suggest a mechanism to suppress transposon mobility that involves m^6^A RNA methylation and sequestration of transposon RNA in SGs. The presence of active ALKBH9B in SGs would explain the decrease in the amount of m^6^A in SGs that we observed in later stages of stress; however, Fan *et al.* (2023) did not reveal a decrease in the amount of m^6^A in SGs as the biotic stress progresses. Our results showed that a high level of m^6^A correlated with a lower quantity of that mRNA in the cell (Figs 3, 5). To protect the mRNAs from degradation and store them in SGs until the end of stress, ALKBH9B could demethylate the transcripts. Therefore, m^6^A might initially promote mRNA recruitment to SGs, but is then removed to enhance mRNA stability. Our RNA-seq data supported this mechanism in lupin because the level of mRNA *ALKBH9B* significantly increased during hypoxia and decreased after reoxygenation (Fig. 3).

In conclusion, our study identifies two distinct zones within plant SGs with different functions. We also demonstrate a limited impact of m^6^A modification on SG assembly; however, the interplay between m^6^A modification and the overall transcript abundance in the cytoplasm plays a regulatory role in selective mRNA partitioning into SGs.

## Supplementary data

The following supplementary data are available at *JXB* online.

Fig. S1. Distribution of poly(A) RNA and PAB2 in meristematic cells of lupin roots under normoxia, hypoxia, and cycloheximide treatment.

Fig. S2. Distribution of poly(A) RNA and PAB2 in a non-meristematic cell of a lupin root under hypoxia.

Fig. S3. Ultrastructure of a lupin root cell under normoxia conditions, and identification of SGs.

Fig. S4. Identification of SGs by immunolocalization with anti-PAB2 antibodies following enrichment of SGs.

Fig. S5. Localization and quantitative analysis of *26S* and *18S* rRNA by FISH in meristematic cells of lupin roots in normoxia and after 15 h of hypoxia.

Fig. S6. Principal component analysis of the RNA-seq data and hierarchical gene clustering.

Fig. S7. GO terms analysis for up- and down-regulated genes in comparisons of hypoxia versus normoxia, reoxygenation versus hypoxia, and reoxygenation versus normoxia.

Fig. S8. Localization by FISH analysis of *HUP7*, *L37*, and *L44* transcripts in meristematic cells of lupin roots under normoxia and hypoxia.

Fig. S9. Localization by FISH analysis of *PCO1* and *WIN1* in meristematic cells of lupin roots under normoxia and hypoxia.

Fig. S10. Control reactions for determination of m^6^A localization in lupin and Arabidopsis root meristematic cells.

Fig. S11. Localization of poly(A) RNA and m^6^A on resin sections in root meristematic cells of Arabidopsis in hypoxia conditions.

Dataset S1. An *ab initio* assembly of the lupin transcriptome with RNA-seq data (GTF format).

Table S1. The sequences of the antisense DNA probes and the primers used in qPCR reactions.

Table S2. Annotation of the *ab initio* transcriptome with results of BLASTP search against SwissProt proteins and orthologs in Arabidopsis obtained from ENSEMBL Plants 55.

Table S3. Results of differential expression analysis from DESeq 2 for the comparisons hypoxia versus normoxia, reoxygenation versus normoxia, and reoxygenation versus hypoxia.

eraf046_suppl_Supplementary_Figures_S1-S11_Table_S1

eraf046_suppl_Supplementary_Table_S2

eraf046_suppl_Supplementary_Table_S3

eraf046_suppl_Supplementary_Dataset_S1

## Data Availability

The RNA-seq data are available in the Gene Expression Omnibus database (https://www.ncbi.nlm.nih.gov/geo/query/acc.cgi?acc=GSE272430) under the accession number GSE272430.

## Abbreviations

ADH1: alcohol dehydrogenase 1
FISH: fluorescence *in situ* hybridization
HRA1: hypoxia response attenuator 1
HUP7: Hypoxia-response Unknown Protein 7
L37: 60S ribosomal protein L37
L44: 60S ribosomal protein L44
m^6^A: N^6^-methyladenosine
PAB2: , Poly(A) Binding Protein 2
PCO1: , Plant Cysteine Oxidase 1
PDC1: Pyruvate Decarboxylase 1
RPB1: DNA-directed RNA Polymerase II subunit
SGs: stress granules
WIN1: ethylene-responsive transcription factor WIN1
