# Supplementary material for: Impact of m6A modification and transcript quantity on mRNA composition in plant stress granules under hypoxia
Source: J Exp Bot. 2025 Feb 5;76(8):2338–55. doi: 10.1093/jxb/eraf046 (PMC12116189; doi:10.1093/jxb/eraf046)
Supplement: eraf046_suppl_Supplementary_Figures_S1-S11_Table_S1 [file eraf046_suppl_supplementary_figures_s1-s11_table_s1.pdf]

SUPPLEMENTARY MATERIALS

FOR

Impact of m^6A Modification and Transcript Quantity on mRNA Composition in Plant Stress Granules

Supplementary figures and tables

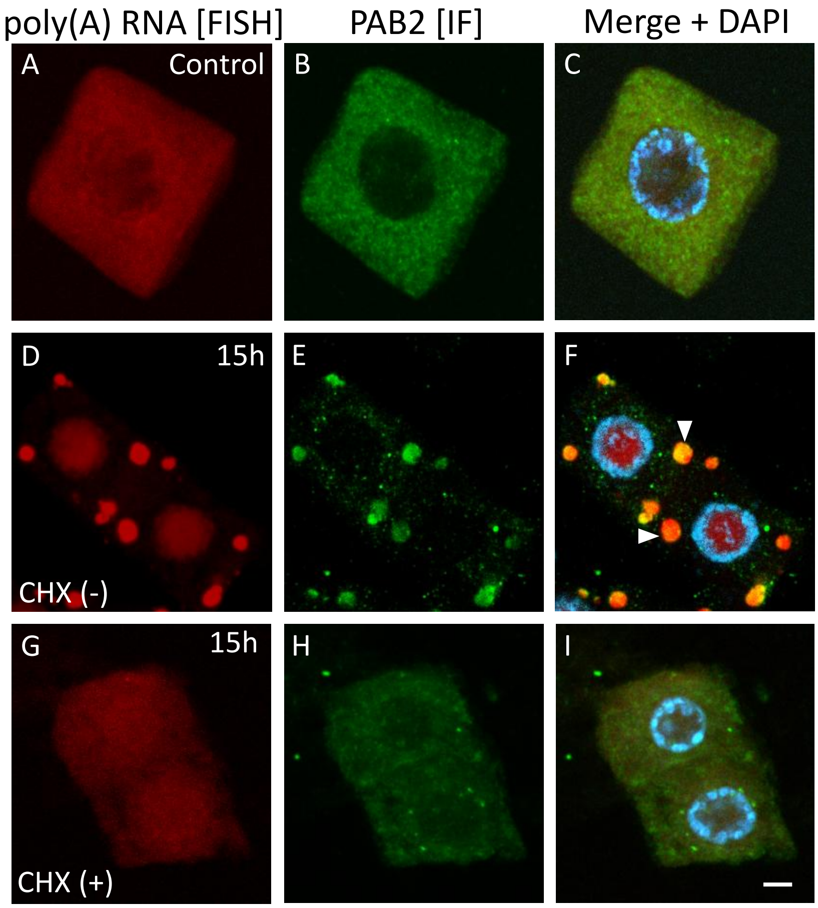

Fig. S1 Distribution of poly(A) RNA (red fluorescence) and SGs marker protein PAB2 (green fluorescence) in meristematic cells of *L. angustifolius* roots in normoxia (**A**-**C**), 15 h of hypoxia (**D**-**F**) and 15 h hypoxia with cycloheximide treatment (**G**-**I**), the arrowheads indicate SGs, merge of signals and DAPI staining (**C**, **F**, **I**), bar 10 µm, FISH - Fluorescence in situ hybridization, IF – immunofluorescence.

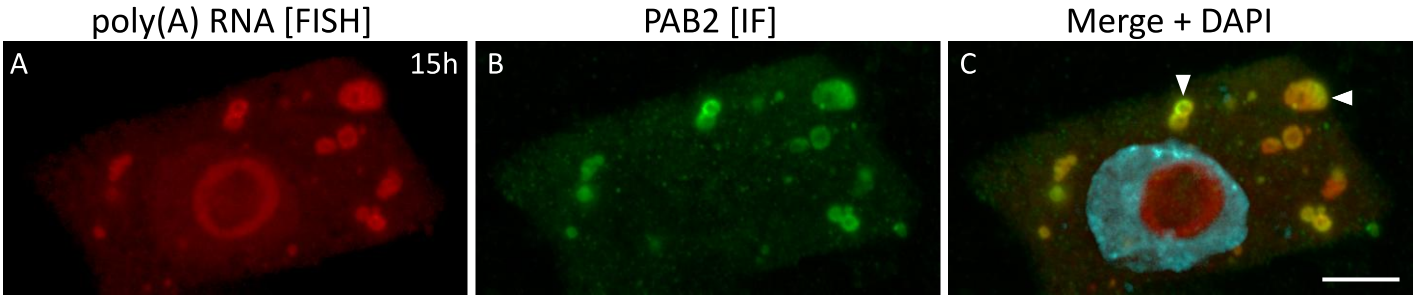

Fig. S2 Distribution of poly(A) RNA (red fluorescence) (**A**) and SGs marker protein PAB2 (green fluorescence) (**B**) in differentiated cell of *L. angustifolius* roots in 15 h of hypoxia, the arrowheads indicate SGs, merge of signals and DAPI staining (**C**), bar 10 µm, FISH - Fluorescence in situ hybridization, IF – immunofluorescence.

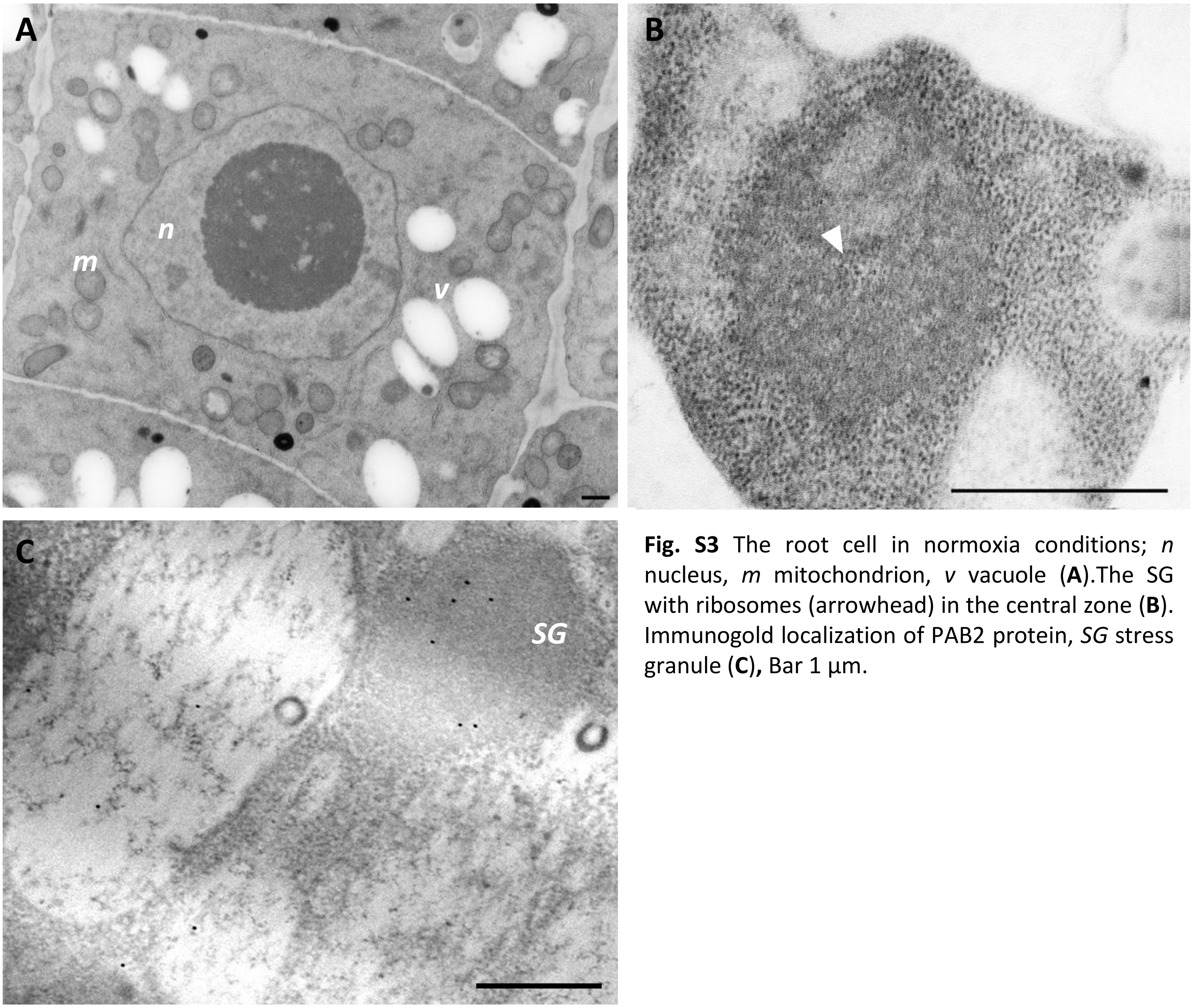

Fig. S3 The root cell in normoxia conditions; *n* nucleus, *m* mitochondrion, *v* vacuole (**A**).The SG with ribosomes (arrowhead) in the central zone (**B**). Immunogold localization of PAB2 protein, *SG* stress granule (**C**)**,** Bar 1 µm.

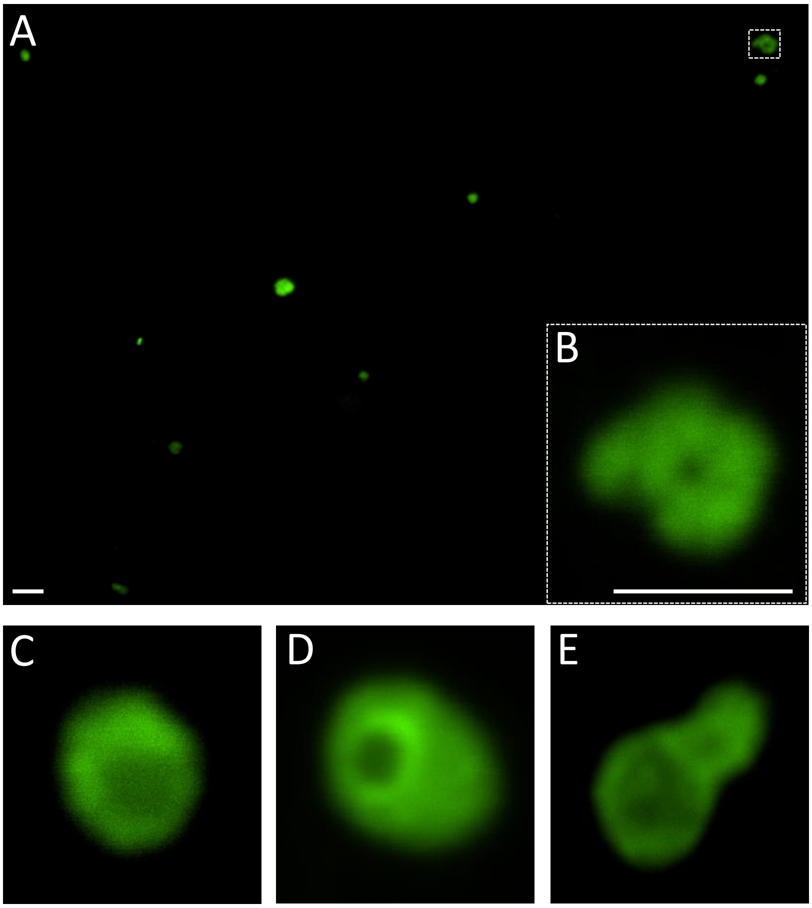

Fig. S4 Identification of SGs by immunolocalization with anti-PAB2 antibodies in SGs enriched fraction (**A-E**), bar 4 µm.

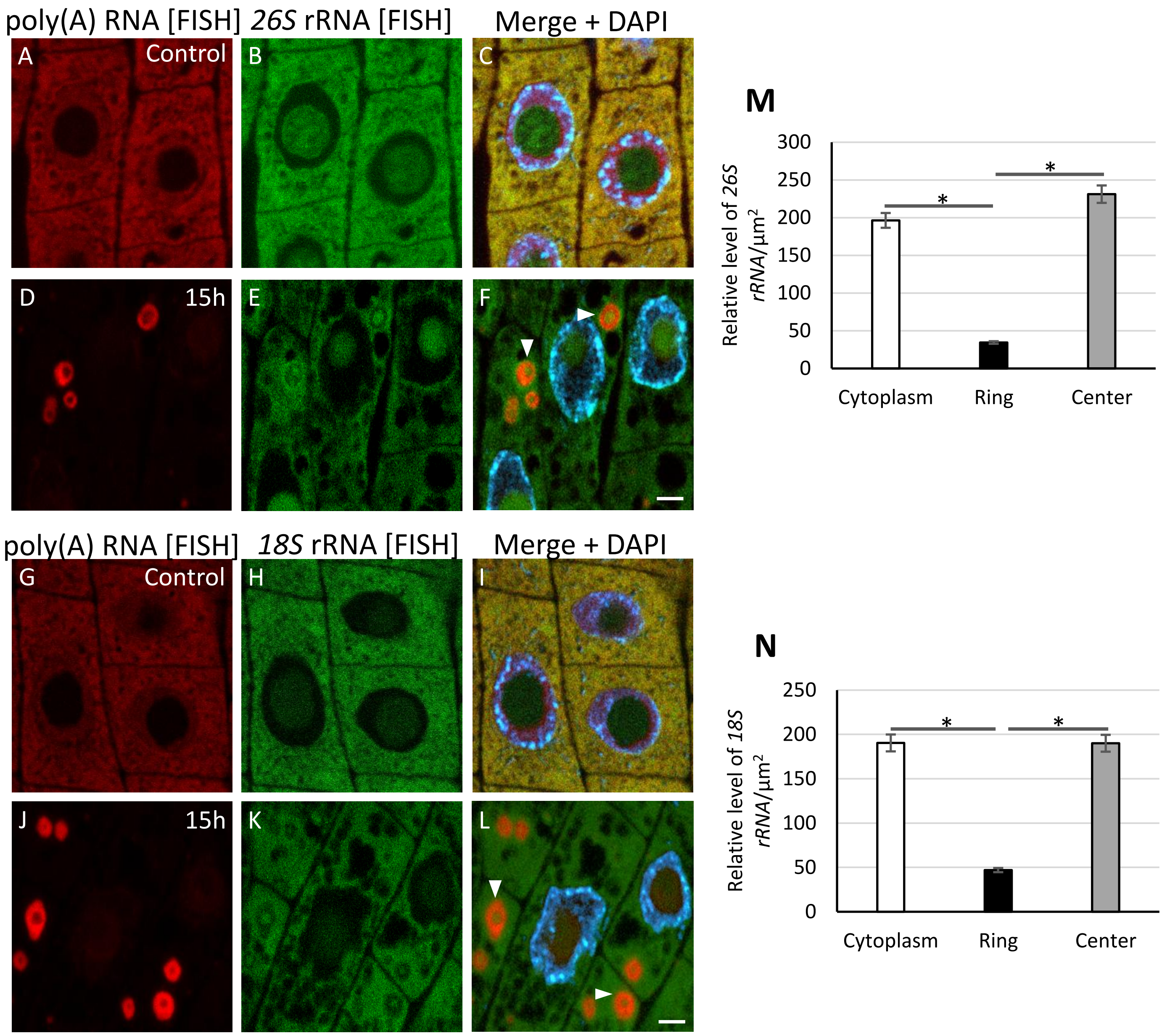

Fig. S5 Localization of poly(A) RNA (red fluorescence) and *26S*, *18S* rRNA (green fluorescence) in meristematic cells of *L. angustifolius* roots in normoxia (**A-C**, **G-I**) and 15 h of hypoxia (**D-F**, **J-L**), merge of signals and DAPI staining (**C**, **F**, **I**, **L**), the arrowheads indicate SGs, bar 10 µm, FISH - Fluorescence in situ hybridization. Quantitative analysis of 26S (**M**) and 18S (**N**) rRNA during hypoxia in the cytoplasm, and in two zones of SGs: ring and central area. Asterisks indicate statistically significant differences (* $P < 0.01$).

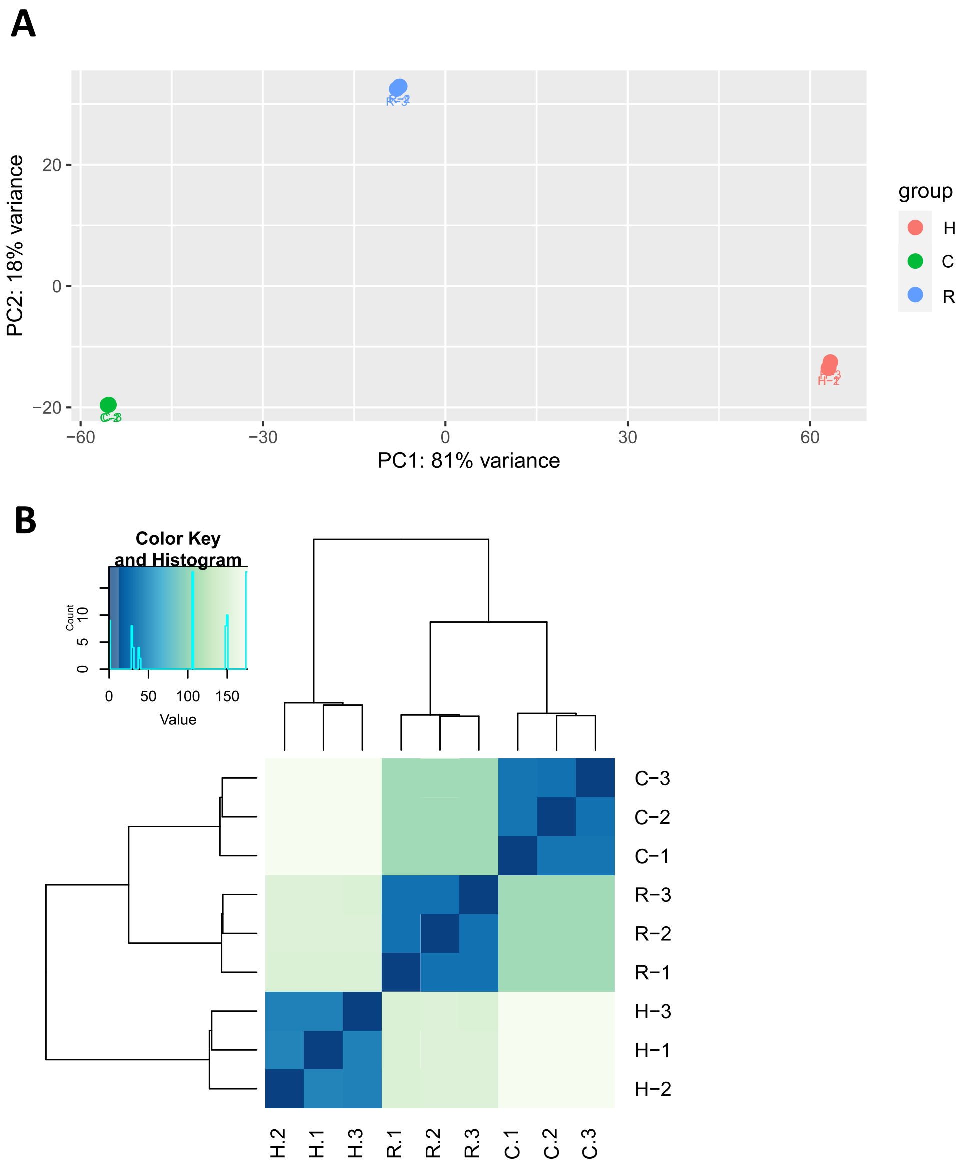

Fig. S6 Principal component analysis (PCA) of the RNA-seq data representing the clustering of biological replicates based on gene expression levels of *L. angustifolius* roots in normoxia (C), hypoxia (H) and reoxygenation (R) conditions (**A**). Hierarchical gene clustering based on the Euclidean distance matrix between the replicates of above samples (**B**).

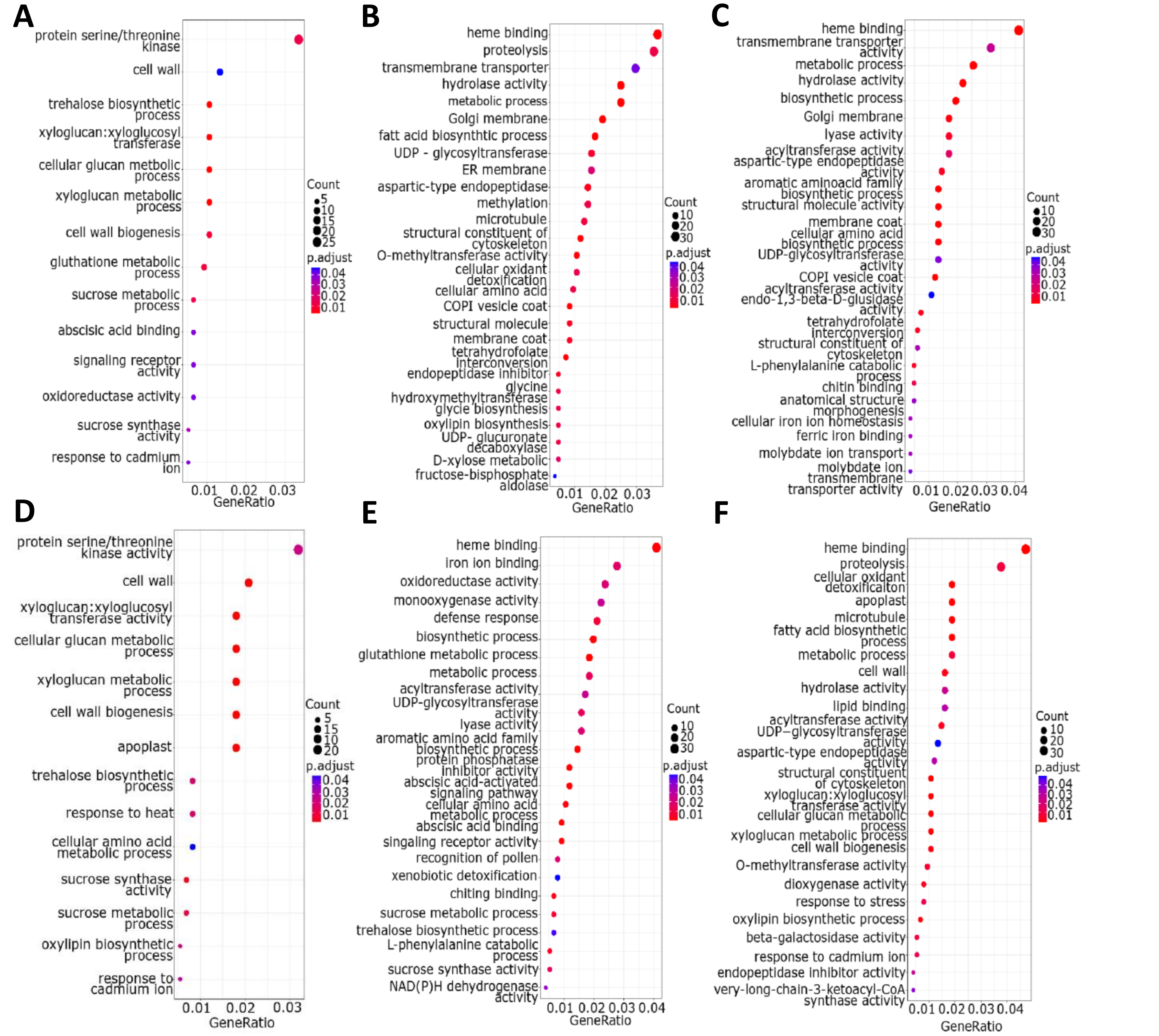

Fig. S7 A dotplot representation of Gene Ontology terms overrepresented among up- (**A, C, E**) and down- (**B, D, F**) regulated genes in hypoxia vs normoxia (**A, B**), reoxygenation vs hypoxia (**C, D**), reoxygenation vs normoxia (**E, F**) comparisons respecitvely.

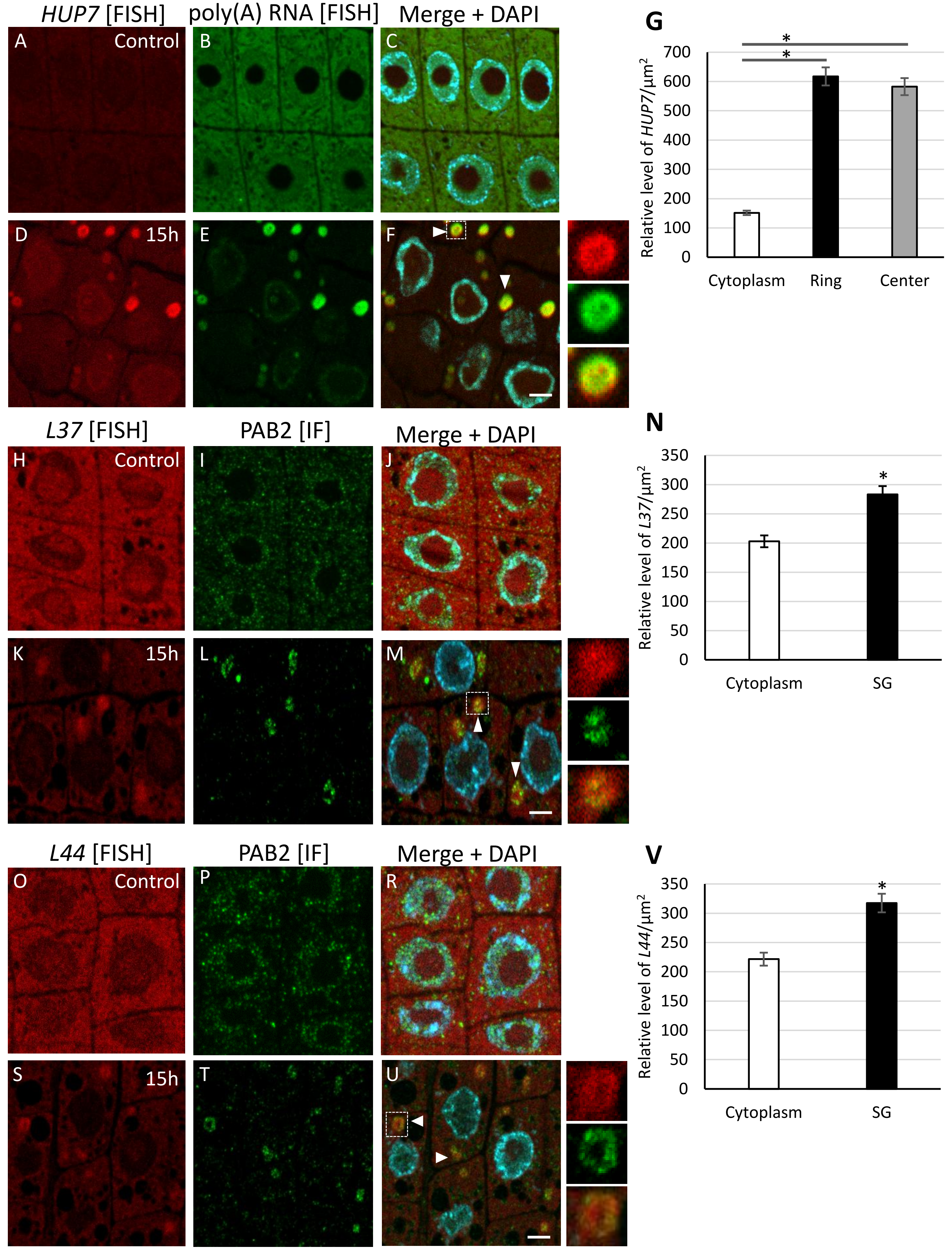

Fig. S8 Localization of *HUP7* mRNA (red fluorescence) and poly(A) RNA (green fluorescence) (**A-F**), *L37* mRNA (red fluorescence) and SGs marker protein PAB2 (green fluorescence) (**H-M**), *L44* mRNA (red fluorescence) and PAB2 (green fluorescence) (**O-U**) in meristematic cells of *L. angustifolius* roots in normoxia (**A-C**, **H-J**, **O-R**) and 15 h of hypoxia (**D-F**, **K-M**, **S-U**), the right-hand panel represents the magnification of SG which is marked with a square, merge of signals and DAPI staining (**C**, **F**, **J**, **M**, **R**, **U**), bar 10 µm, FISH - Fluorescence in situ hybridization, IF – immunofluorescence. The relative fluorescence intensity of: *HUP7* mRNA in the cytoplasm, ring and central area of SGs (**G**); *L37* mRNA (**N**), *L44* mRNA (**V**) in the cytoplasm and SGs of roots cells subjected 15 h to hypoxia stress.

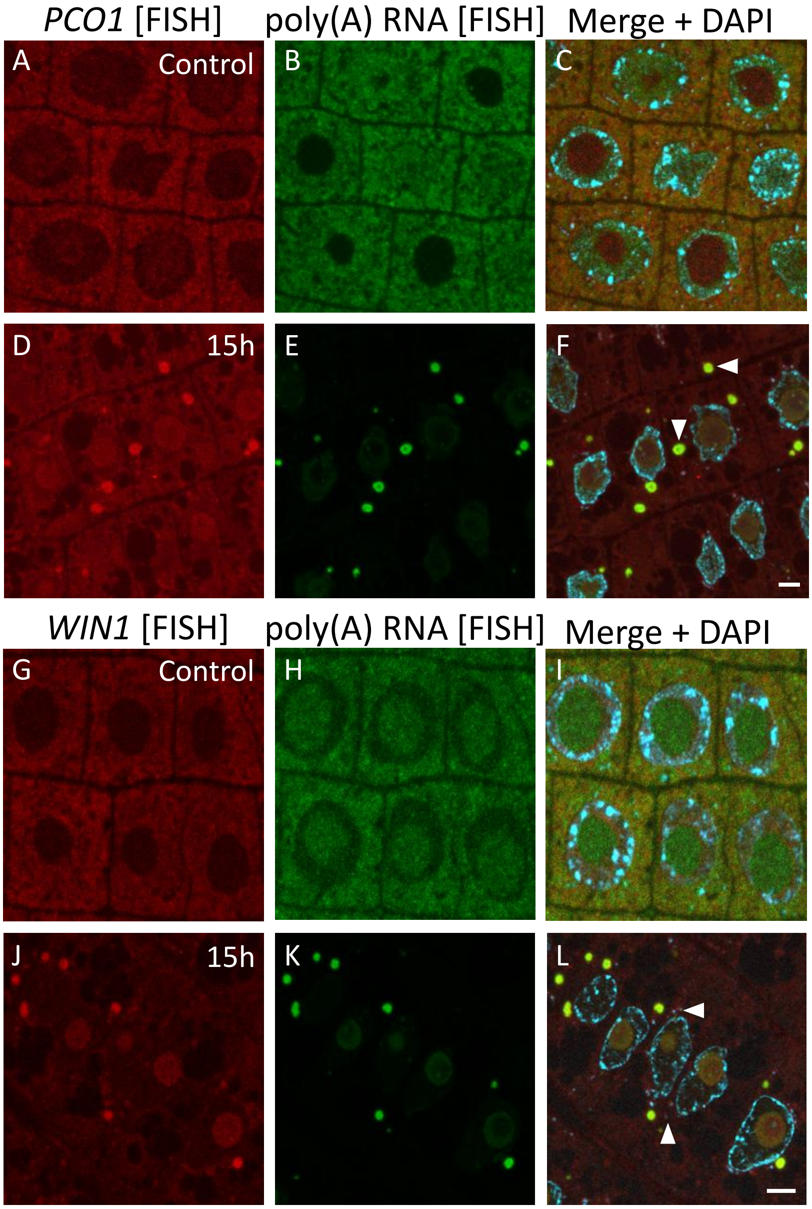

Fig. S9 Detection of *PCO1* transcripts (red fluorescence) and poly(A) RNA (green fluorescence) (**A-F**), *WIN1* mRNA (red fluorescence) and poly(A) RNA (green fluorescence) (**G-L**) in meristematic cells of *L. angustifolius* roots in normoxia (**A-C**, **G-I**) and 15 h hypoxia (**D-F**, **J-L**), the arrowheads indicate SGs, merge of signals and DAPI staining (**C**, **F**, **I**, **L**), bar 10 µm, FISH - Fluorescence in situ hybridization.

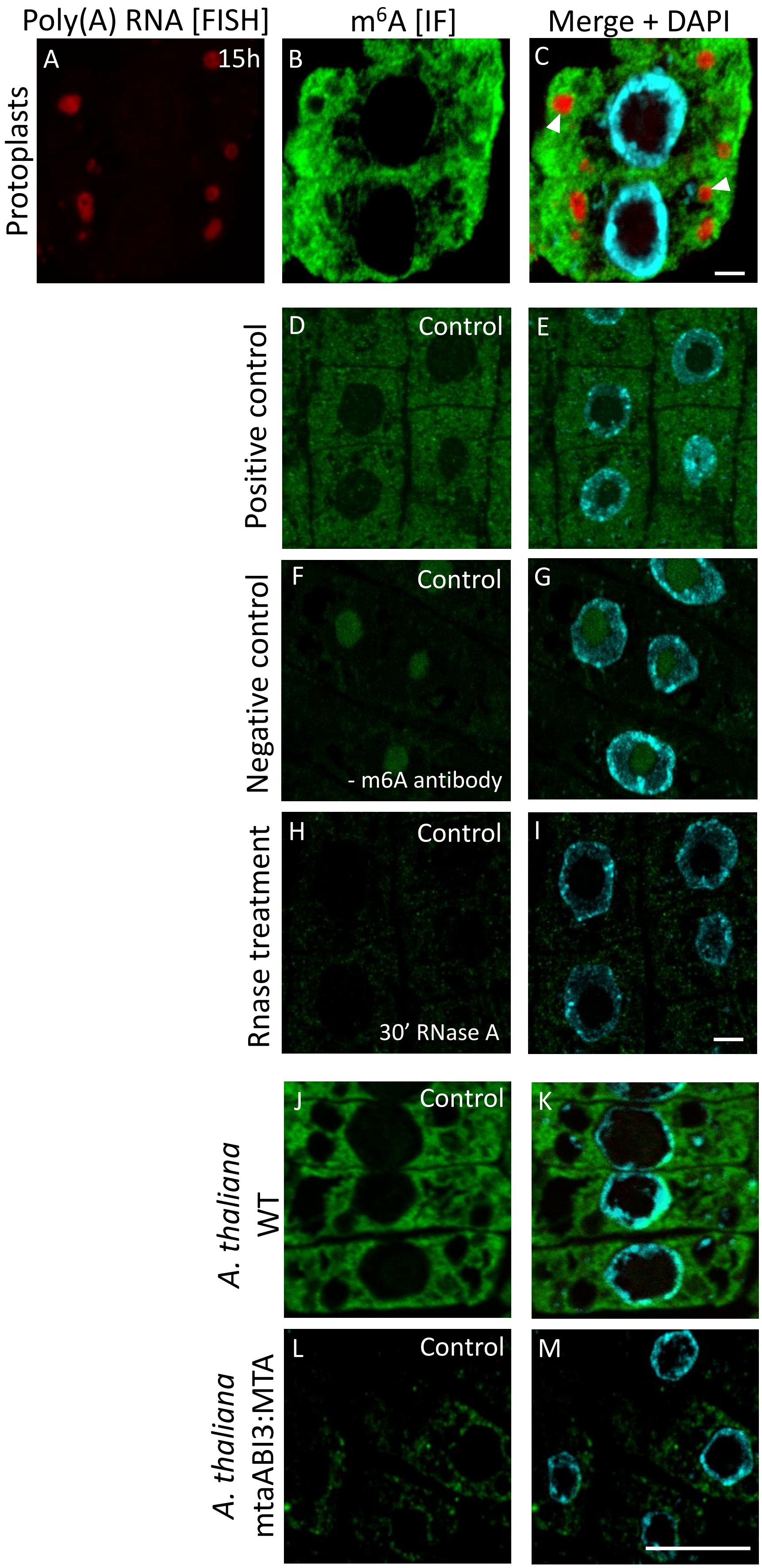

Fig. S10 Localization of m^{6}A in protoplasts of lupin root meristematic cells in normoxia (**A**-**C**). Control reaction of m6A localization in lupin root meristematic cells in normoxia (**D**-**E**), omission of primary antibodies results in no signal, indicating antibody specificity for m6A (**F**-**G**), treatment with RNase A before immunofluorescence leads to disappearance of antibody fluorescence signal for m^{6}A (**H**-**I**). Immunofluorescence localization of m^{6}A in root cells of *A. thaliana* wild-type (**J**-**K**) and mtaABI3:MTA mutant with reduced levels of m6A (**L**-**M**), merge of signals and DAPI staining (**C**, **E**, **G**, **I**, **K**, **M**), bar 10 µm, FISH - Fluorescence in situ hybridization, IF - immunofluorescence.

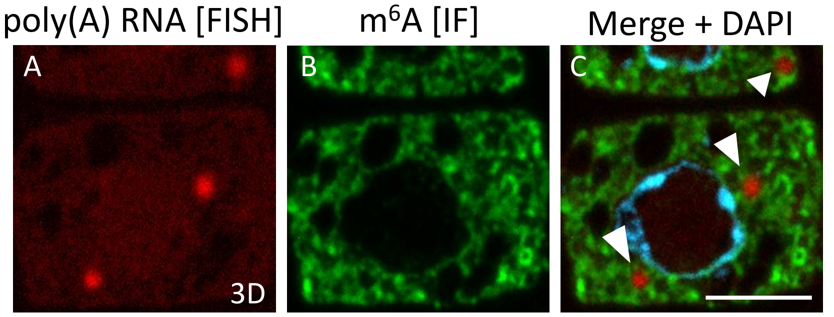

Fig. S11 Localization of poly(A) RNA (red fluorescence) (**A**) and m^{6}A (green fluorescence) (**B**) on resin sections in meristematic cells of *A. thaliana in* 3 days hypoxia conditions, the arrowheads indicate SGs, merge of signals and DAPI staining (**C**), bar 5 µm, FISH - Fluorescence in situ hybridization, IF – immunofluorescence.

Tab. S1 The sequences of antisense DNA probes (**A**), sequence of primers used in qPCR reaction (**B**)

A

DNA probe	Sequence
ADH1-1	CACCTGGTTTCAGATGAGTCACACCCTCTCCTA
ADH1-2	GAAATCCTTGCCCCTTCAGCAGCAGCAAGGCC AACA
ADH1-3	CCTATCAGTGTTGATCCTAAGAAGATC
HUP7	TTGGCTAGTCGAGCTGTGTTGCCATGTGATAC AA
PCO1	ATCAGCTAAAGTCCTCTCAGTACCCATTCAA
WIN	ACTGTGATGGTTGCTTGTTTTGTTGTTATCAGA
RPB1	CATAATCATCTTTTGTAAAGTGAGGCAATGTCC GGTCTAT
L37	TGATAATGAAGCCACTAAATATCATAGGAA
L44	GTGCAGTGTATGCTTCTTGCATTCCTTGTTC
18s rRNA	TTATCTAATAAATGCATCCTTCCAGGAAGTCG
26s rRNA	TCCCGACAGGACGCTCTCACTCGAACCCTTC

B

Starter	Starter sequence 5'⟶3'
ADH1-1	F: ATGAAGCTGGAGGGATTGTG<br>R: AGGTTCCGACAAAATGATGC
ADH1-2	F: CATGAAGCTGGAGGGATTGT<br>R: CGAGGTTCCGACAAAATGAT
UBC5	F:GAAATCGAGCGATGAAGAGC<br>R: CCCCTACCAGCAGCAATAAA
L37-1	F: AGGGTTCTGCATCTGCATCT<br>R: ACGACTCTTCTGGAGGTGGA
L37-2	F:ATGGGGAAGGGAACAGGTA<br>R: ACGAATTGCCTTCACACTCC
L44-1	F: GCAAACAGTCCGGTTATGGT<br>R: CCCTTCTTGTCACCACCAAT
L44-2	F: TGCAAGAACAAGGAATGCA<br>R: GCACTGCAACCTCAAGACAA
RPB1-1	F: TTGGATTGAAACCCAGAAGC<br>R: TTCAGCCTCAAACACACTGC
RPB1-2	F: TACCCCGAGACTGTGACTCC<br>R: ATGACGCTCCACCTTGTACC
HUP7-1	F:CACGTCATTCCAAGAGCGAG<br>R:TCGTACATCAAATGCGCTGG
HUP7-2	F:CCGCCGCTGAAATCTTGTAA<br>R:CCCCAAATGGCAAGGAAGAG
WIN-1	F:CGTGCAGGGTCTAGTTCTGA<br>R:TGCTCCTCATCCAACCCATT
WIN-2	F:GATGAGTGGAAGAAACGCCA<br>R:TCAGAACTAGACCCTGCACG
PCO1-1	F:CGCTGACTCTTCCACCTTTG<br>R:GAGGACCAAGCACGTCTAGA
PCO1-2	F:AACCGGTGGCGTACTAAGAA<br>R:ATACGGCATGTCAGGTGTCA
